# Genotype Diversity, Wild Bird-to-Poultry Transmissions, and Farm-to-Farm Carryover during the Spread of the Highly Pathogenic Avian Influenza H5N1 in the Czech Republic in 2021/2022

**DOI:** 10.3390/v15020293

**Published:** 2023-01-20

**Authors:** Alexander Nagy, Martina Stará, Lenka Černíková, Lada Hofmannová, Kamil Sedlák

**Affiliations:** State Veterinary Institute Prague, Sídlištní 136/24, 165 03 Prague, Czech Republic

**Keywords:** H5N1, HPAI, avian influenza, highly pathogenic avian influenza, outbreak, poultry

## Abstract

In 2021/2022, the re-emergence of highly pathogenic avian influenza (HPAI) occurred in Europe. The outbreak was seeded from two sources: resident and reintroduced viruses, which is unprecedented in the recorded history of avian influenza. The dominant subtype was H5N1, which replaced the H5N8 subtype that had predominated in previous seasons. In this study, we present a whole genome sequence and a phylogenetic analysis of 57 H5N1 HPAI and two low pathogenic avian influenza (LPAI) H5N1 strains collected in the Czech Republic during 2021/2022. Phylogenetic analysis revealed close relationships between H5N1 genomes from poultry and wild birds and secondary transmission in commercial geese. The genotyping showed considerable genetic heterogeneity among Czech H5N1 viruses, with six different HPAI genotypes, three of which were apparently unique. In addition, second-order reassortment relationships were observed with the direct involvement of co-circulating H5N1 LPAI strains. The genetic distance between Czech H5N1 HPAI and the closest LPAI segments available in the database illustrates the profound gaps in our knowledge of circulating LPAI strains. The changing dynamics of HPAI in the wild may increase the likelihood of future HPAI outbreaks and present new challenges in poultry management, biosecurity, and surveillance.

## 1. Introduction

Highly pathogenic avian influenza (HPAI) poses a significant threat to the poultry industry worldwide. In the last decade, three major outbreaks of H5Nx HPAI have occurred in Europe, in 2016/2017, 2020/2021 and 2021/2022 [1]. The latest pan-European HPAI outbreak in 2021/2022 affected 33 EU/EEA countries and the UK, with more than 5200 virus detections. Of these, as of June 2022, 2398 cases were related to poultry establishments. This resulted in the culling of at least 46 million birds, marking the recent outbreak as one of the worst ever recorded in Europe [1]. 

The dominant subtype of the European HPAI 2021/2022 outbreak was H5N1, which contrasts with previous HPAI seasons, with the H5N8 subtype as the most prevalent [2,3]. The H5 hemagglutinin (HA) of the 2021/2022 viruses belonging to the 2.3.4.4.b clade of the A/goose/Guangdong/1/1996 H5N1(gs/GD/96) lineage.

The origin of the European H5N1/2021 HPAI strains can be traced to the north-west of the Netherlands, where avian influenza virus of the H5N1 subtype with high pathogenicity was detected on 16 October 2020 in four living Eurasian wigeons (Mareca penelope) sampled in a duck decoy in Hippolytishoef [4]. This virus apparently co-circulated with A/chicken/Iraq/1/2020-like H5N8 (A/ck/Iq/1/2020) strains [5], which subsequently became prevalent from November 2020 and caused the second pan-European HPAI outbreak in 2020/2021 [6]. Genotype analysis of the Dutch H5N1 strains (A/eurasian wigeon/Netherlands/1, 4 and 5/2020 strains, commonly referred to as A/ew/NL/2020) revealed that they arose from a 2:6 reassortment with the retention of the “Iraqi” H5 and MP segments on the backbone of the unknown Eurasian low pathogenic avian influenza (LPAI) viruses from 2019–2020 [5,6], apparently including an HxN1 subtype. 

Longitudinal reports show that between November 2020–May 2021, A/ew/NL/2020-like H5N1 strains were sporadically detected in wild and captive birds across Europe and caused several outbreaks in poultry [6,7,8]. However, since April 2021, the frequency of H5N1 HPAI detections has started to increase [8]. This trend continued during June-September 2021, despite an overall decrease in avian influenza incidence throughout the summer months [9]. Taken together, the data suggests that the H5N1 HPAI has circulated at the background level throughout the entire period of dominance of the A/ck/Iq/1/2020-like H5N8 strains. Moreover, one subclade apparently persisted during the summer in the wild bird population in northern Europe [9,10]. Genotyping of the H5N1 strains collected from 2020/2021 indicated the wide distribution of the original A/ew/NL/2020-like genotype constellation, with reassortants having been observed since the end of September 2021 [11]. 

However, from the beginning of October 2021, the incidence of H5N1 HPAI started to increase, which subsequently resulted in the 2021/2022 pan-European H5N1 HPAI outbreak [12]. Comprehensive phylogenetic analysis of H5 HA sequences suggested that this outbreak was seeded from two principal sources: (i) resident viruses maintained in northern Europe throughout the summer of 2021, and (ii) viruses circulated outside Europe showing an African ancestry [10].

In the Czech Republic (CZE), the H5N1 HPAI virus was first detected in backyard poultry in late September 2021, approximately three months after the H5N8 virus had subsided in the country [13]. The last detection of the H5N1 virus was dated to mid-April 2022. 

In the study presented, a whole genome sequence and a phylogenetic analysis of 57 HPAI strains and two LPAI strains of the H5N1 subtype were performed, using collections from wild birds and both backyard and commercial poultry in the Czech Republic during the 2021/2022 season. The data obtained were evaluated in order to elucidate the origin, geographic distribution, and genotype diversity of the circulated strains and to infer relationships between the H5N1 HPAI viruses detected in wild birds and backyard and commercial poultry.

## 2. Materials and Methods

All bird carcasses received were subjected to autopsy. Cloacal or tracheal swabs, or parts of multiple organs, were collected for virus detection and next-generation sequencing. Pooled organs were homogenized in RNA later (Invitrogen) and swabs in PBS. Total nucleic acid was extracted from 200 µL supernatants of pooled organs or swabs (MagNAPure Compact, MagNAPure 24 or MagNAPure 96 instruments, Roche) and eluted into 50 µL. For detection and identification of H5N1, RT-qPCR methods specific for the generic influenza A virus and the H5 and N1 subtypes were performed in combination with cleavage site sequencing [14,15,16,17,18].

Real-time next-generation sequencing was performed using nanopore technology (MinION Mk1B, R9.1.4 flow cells; Ligation sequencing kit and Native barcodes, Oxford Nanopore Technologies, Oxford, UK). The H5N1 genome was amplified with a set of RT-PCR reactions (OneStep RT-PCR Kit, Qiagen, Hilden, Germeny) in a final volume of 12.5 µL (10 µL RT-PCR mix + 2.5 µL total NA extract; primers available on request). Sequencing libraries were purified (SPRIselect beads; Beckman Coulter, Brea, CA, USA) and quantified (QIAxpert; Qiagen). End-preparation, native barcoding, and adapter ligation sequencing were performed according to the manufacturer’s instructions. Basecalling was performed with Guppy v4.40 and demultiplexing and reference mapping by implementing the RAMPART (Read Assignment, Mapping, and Phylogenetic Analysis in Real Time) module of the ARTIC bioinformatic pipeline [19] with the concatenated H5N1 genome as a reference. Consensus sequences were obtained using Samtool’s Mpileup and Bcftools programs [20] and submitted to the GISIAD EpiFlu database with accession codes listed in Table 1. HA subtype numbering conversion, sequence feature inference, and antiviral resistance risk assessment were performed using the corresponding tools of the Influenza Research Database [21].

The Czech H5N1 genomes were compared in the context of other European sequences collected between 2020 and 2022 and stored in the GISAID database. Sequences were aligned using the MAFFT (Multiple Alignment using Fast Fourier Transform) [22]. Alignment trimming and format conversion (Phylip full names and padded) were performed using AliView [23]. Sequence identity matrices and sequence difference count matrices (SIMs and SDCMs) at the nucleic and amino acid levels were calculated using BioEdit 7.0.9.0 [24]. Maximum likelihood (ML) trees (IQ-TREE multicore version 2.0.3 for Linux 64-bit [25]; 1000 replicates) were calculated separately for each genomic segment and also as a species correlation tree constructed from genomic concatenates. Sequence concatenation was performed using the Union program from EMBOSS [26]. For all trees, the best-fitting model was selected according to the Bayesian information criterion (BIC). Phylogenetic dating was inferred using the IQ-TREE program by implementing the least square dating (LSD2) method [27].

## 3. Results

### 3.1. Overview of the 2021/2022 Avian Influenza Season in the Czech Republic

**Figure 1:**
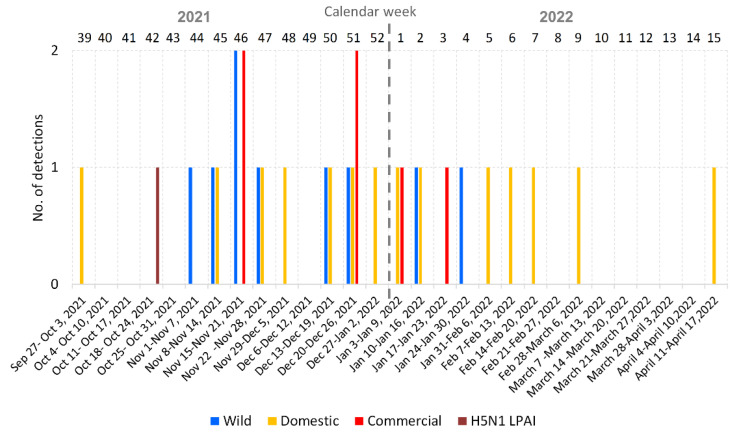
Distribution of AIV detections in the 2021/2022 season in the Czech Republic. Bar chart showing the number of HPAI/LPAI outbreaks detected per calendar week.

In the Czech Republic, the 2021/2022 H5N1 HPAI season started with an outbreak in a backyard poultry flock on 27 September 2021 [11] and lasted until 13 April 2022 (calendar weeks 39–15; Figure 1). Like in the previous season [13], infections in backyard poultry were observed throughout the entire outbreak, while the infections in commercial poultry occurred from November 2021 to January 2022. In wild birds, the H5N1 HPAI virus was detected only sporadically.

The H5N1 HPAI virus was identified during 14 outbreaks in backyard poultry (including chickens, ducks, geese, guinea fowls, and Muscovy ducks) and five outbreaks in commercial farms (Table 1). Further, the H5N1 HPAI virus was detected in 32 carcasses of wild birds collected in nine locations: 13 mute swans (Cygnus olor), four grey herons (Ardea cinerea), four great egrets (Ardea alba), and 11 unspecified bird species. More than 205,000 poultry were destroyed during the management of the H5N1 HPAI outbreak. Finally, on 22 October 2021, H5N1 LPAI was detected in a commercial farm consisting of 4200 geese and 800 broiler poultry. The flock was depopulated. An overview of the macroscopic pathological changes associated with H5N1 HPAI infection is given in Supplementary Material file S1, Table S1.

The most important commercial farms affected by H5N1 HPAI were a goose breeding farm with ~6000 birds in the South Bohemia region (Table 1, IDs with asterisk), a common pheasant (Phasianus colchicus) breeding farm with ~6500 birds in the South Moravia region (Table 1; IDs 25827/21) and a farm with more than 189,000 layers in the Litoměřice region (ID 25690/21). In particular, the HPAI outbreak in breeding geese detected on 18 and 19 November 2021 was the most critical (IDs 22608/21 and 22750/21). On-site veterinary inspection reported tremor as the most remarkable clinical sign of infection accompanied by increased mortality. Postmortal examinations of the involved birds showed a generally good condition, mainly with an enlarged spleen, lung hyperemia, and in some individuals with hemorrhagic diathesis associated with the HPAI infection. Due to the importance of a farm possessing a unique genetic lineage of geese belonging to the national genetic resources base and with more than 50 years of tradition in breeding, an exemption from total depopulation with only selective culling of the RT-qPCR positive birds was granted by the State Veterinary Administration. However, the H5N1 HPAI infection was continuously detected in the affected holding during three additional sampling events, until the last one on 19 December 2021 (Table 1). The remaining birds underwent vaccination. 

### 3.2. Molecular and Phylogenetic Analysis of the Detected AIV Viruses

To gain an insight into the genomic diversity of the H5N1 strains circulating in the Czech Republic in the 2021/2022 season, 57 HPAI viral genomes were sequenced and analyzed. Representative whole genome sequences were obtained from all 9/9 wild bird sampling localities, 10/14 backyard flocks, and 3/5 commercial farms. Overall, genomic sequence information was available from 22/28 (78.6%) of all cases. In addition, two H5N1 genomes were sequenced from the LPAI outbreak. 

All HPAI viruses carried the amino acid motif PLREKRRKR/GLF in the H5 HA cleavage site, whereas the LPAI genomes held the PQREKR/GLF motif. 

All sequenced genomes contained the full-length PB1-F2 gene, and both NS alleles were present in the data pool. No deletions were observed in the N1 stem region, suggesting no adaptation in backyard poultry [28]. Naturally occurring substitutions associated with increased affinity for human-type receptors (H3 numbering S137A, T160A, S239P) [29,30,31] were present in HAs of all genomes, including H5N1 LPAI, as well as mutations in the PB2 (L89V, G309D, T339K, T477G, I495V, A676T) and M1 (N30D and T215A) genes, conferring enhanced replication in mammalian cells [32,33]. In addition, a N319/K substitution in the NP gene [34] was observed in HPAI strains from pheasants (ID 25827/21).

Phylogenetic analysis of the H5 sequences showed that all CZE/2021-2022 HPAI strains belonged to the A/ck/Iq/1/2020-like clade within the 2.3.4.4b lineage. The CZE/2021 LPAI showed a Eurasian low-pathogenic H5 origin, with the closest relationship to the H5N1 LPAI strain detected in a mallard in Italy in 2021 (Supplementary Material file S1, Figure S1). The close relationships between the H5 sequences from the Czech poultry and wild birds suggest frequent transmissions from the wild bird reservoir.

**Figure 2:**
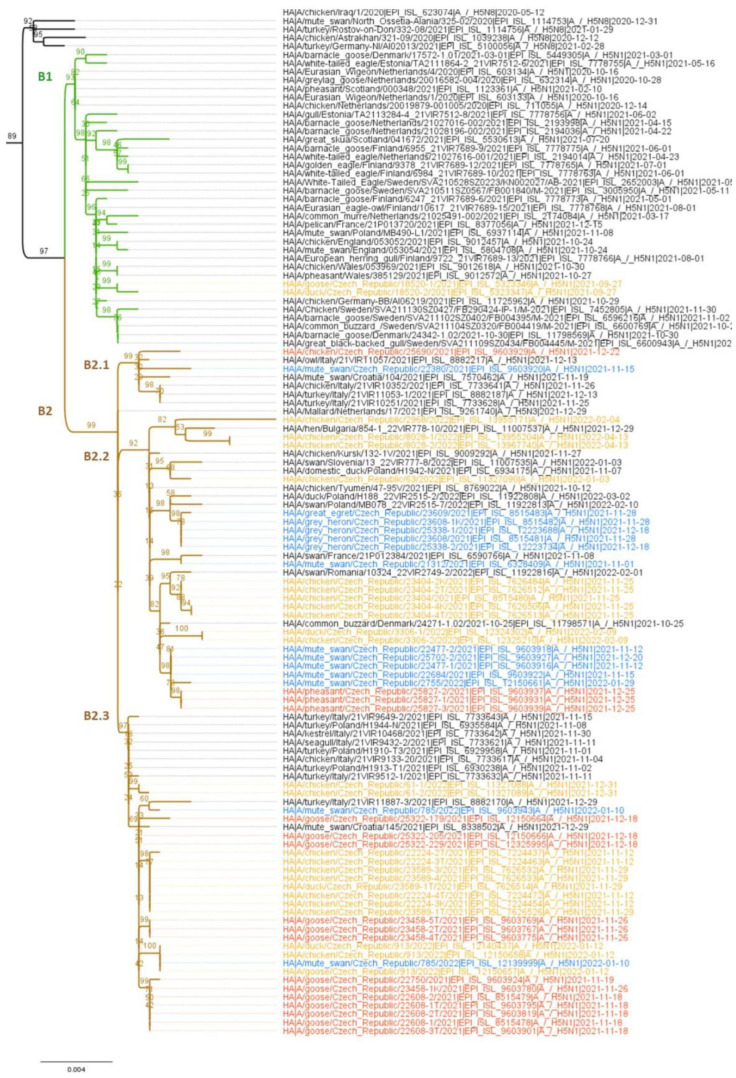
Phylogenetic analysis of H5 hemagglutinin. Species correlation ML tree consisting of Czech and selected Eurasian H5 HPAI sequences collected during 2021–2022 and rooted to A/guinea fowl/Nigeria/2019 H5N8 HA. The tree was calculated by the IQ-TREE program implementing TN + F + I as the best-fitting model selected according to the Bayesian information criterion. The tree was drawn to scale, with branch lengths measured in the number of substitutions per site. For each branch, bootstrap values (1000 replicates) were given in percentages. Subclades B1 and B2 were colored green and brown, respectively. The virus taxa of the H5N1 strains sequenced in the present study were highlighted according to origin: red-commercial; orange-backyard; and blue-wild. A more comprehensive H5 tree is provided in Supplementary Material file S1, Figure S1.

In a recent study on the origin of European 2021/2022 HPAI viruses, Pohlman et al. (2022) identified two main sources of reintroduction: (i) the re-emergence of resident strains that persisted in Europe throughout the summer of 2021 (H5 clade B1), and (ii) the incursion of divergent strains showing a wider distribution with African ancestry (H5 clade B2) [10]. Putting our data into context with this finding revealed the presence of the Czech H5N1/2021–2022 strains in both H5 clades (Figure 2). All but one HPAI virus belonged to the clade B2 encompassing reintroduced viruses. Here, subdivision into at least three well-supported subclades, B2.1–B2.3, is evident. In contrast, the remaining HPAI virus (IDs 18520-1,2/21), was included in the clade B1, consisting of resident European H5N1 viruses. Interestingly, IDs18520-1,2/21 represent the index virus of the 2021/2022 season in the Czech Republic and were among the first recognized genotype alterations since the emergence of H5N1 HPAI in 2020 [11].

### 3.3. Genotype Diversity of the Czech H5N1 2021/2022 Viruses

**Figure 3:**
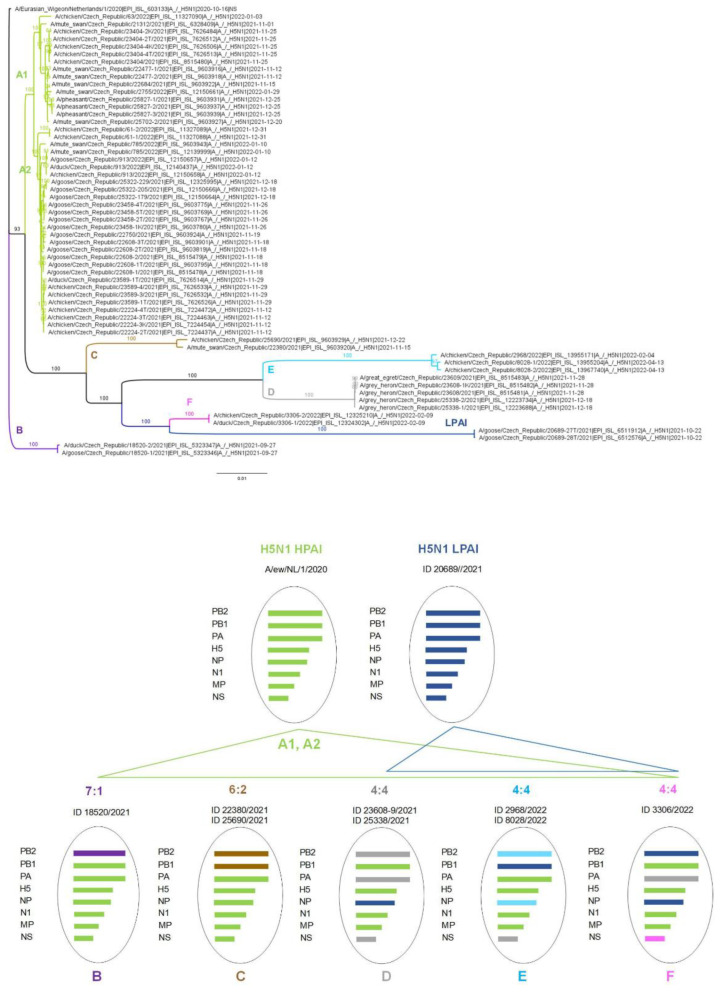
Genotyping of Czech H5N1 HP/LPAI strains detected during the 2021/2022 influenza season. The ML tree was calculated based on 59 concatenated genomes and rooted to A/ew/NL/1/2020 H5N1. The order of segments in the concatenated genomes is PB2, PB1, PA, H5, NP, N1, MP, and NS. The tree was computed using IQ-TREE (GTR + F + R2 as the best-fitting model selected according to the Bayesian information criterion) and scaled with branch lengths measured in the number of substitutions per site. For each branch, bootstrap values (based on 1000 replicates) were given in percentages. Three branches corresponding to discrete genotypes were labeled as A–F and LPAI and highlighted with a genotype-specific color. Further, the figure summarizes the segment constellations of the identified influenza virus genomes inferred from SIMs, SDCMs, and phylogenetic trees calculated individually for each segment (Supplementary Material file S1, Figure S1 and Supplementary Material file S2).

ML trees calculated for all genomic segments revealed considerable genetic variability among Czech H5N1 strains (Figure 3; Supplementary Material file S1, Figure S1). Based on phylogenetic divergences and the SIM threshold empirically set to ≤97% (Supplementary Material file S2), six different HPAI genotypes were recognized. 

Most H5N1 strains retained the A/ew/NL/2020-like genomic constellation, referred to as genotype A. Here, bifurcation into two well-supported subclades, A1 and A2, is evident. Interestingly, genomic subclades A1 and A2 (Figure 3) correspond to H5 subclades B2.2 and B2.3 (Figure 2), suggesting two main incursion events.

Genotype A apparently served as the ancestor for all other genomic constellations, as all genotypes contained at least half of the genomes from genotype A. Genotype B, the only H5N1 virus belonging to the resident H5 subclade B1, arose from a 7:1 reassortment by swapping its PB2 segment with Omsk/2018/H3N8 or Novosibirsk/2019/H3N8-like LPAI strains (Supplementary Material file S1, Figure S1). Similarly, reassortment at a 6:2 ratio by exchanging PB2 and PB1 segments with Novosibirsk/2018/H12N5 and Novosibirsk/2020/H12N5-like LPAI viruses resulted in genotype C. In addition, the H5 HA of genotype C belonged to the distinct subclade B2.1 of the reintroduced viruses (Figure 2).

The reassortment patterns of genotypes D, E, and F are not clear. Here, the 4:3:1 and 4:2:1:1 segment ratios (Figure 2) allow for different scenarios. However, the presence of the H5 HAs exclusively within the B2.2 subclade suggests an ancestral role for the A1 genotype. Furthermore, the analysis revealed the common origin of the NP segment of genotype D, PB1 of genotype E, and PB2 and NP of genotype F with corresponding segments of the co-circulating LPAI H5N1 strain ID 20689/22 (Figure 3, Supplementary Material file S1, Figure S1). Although the degree of ancestry varied and the segments in question are shared with other LPAI subtypes, our data suggests that Czech H5N1 LPAI-like viruses played a significant role in the genomic diversification of the HPAI genomes. This is particularly evident from the closest relationships between the NP segment of genotype F (ID 3306/22) and CZE 20689/21 LPAI (nucleotide sequence identity of 99.4%; eight nucleotide and one amino acid changes; Supplementary Material file S2), suggesting a very recent reassortment event. Finally, the phylogenetic patterns support a second-order 6:2 reassortment between genotypes D and F, involving an H5N1 LPAI-like (PB2) segment in combination with an alternative NS allele.

### 3.4. Molecular Epidemiology of the Czech H5N1 2021/2022 Viruses

**Figure 4:**
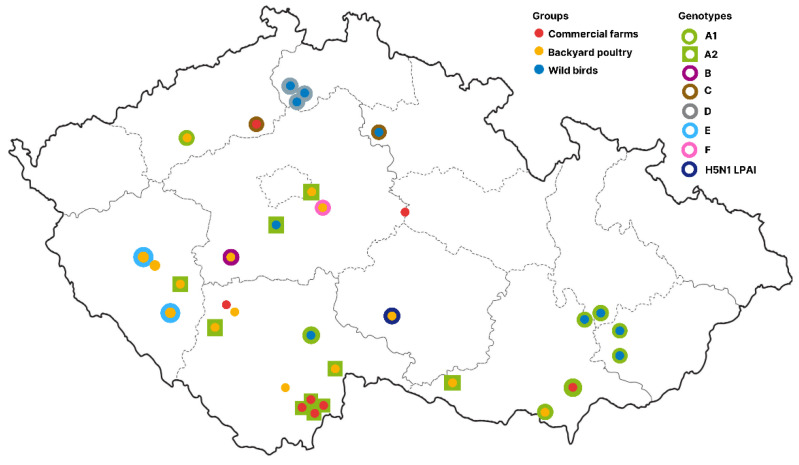
Geographical distribution of H5N1 genotypes. The figure shows the localities of H5N1 virus detections in the 2021/2022 season on a blind map of the Czech Republic. Solid dots indicate bird categories (wild, backyard, and commercial). Localities with available genotype information are surrounded by a halo indicating genotype assignment according to Figure 3. An interactive version of the map is available online at https://www.google.com/maps/d/u/0/edit?mid=1_pWrJqfuItU2UwgIuwHjJDsG-tugjU4q&usp=sharing (accessed on 20 November 2022).

The map in Figure 4 shows the geographical distribution of all H5N1 genotypes identified in the Czech Republic during the 2021/2022 season. The index case, ID 18520/21, is the only known representative of the resident B1 lineage among the detected viruses [10]. This finding was followed by an outbreak of H5N1 LPAI in commercial geese with a close phylogeny to other European H5N1 viruses of low pathogenicity found mainly in Italy.

As can be seen, genotype A was the most widespread in the southern regions of the Czech Republic. Here, the cluster of genotype-A1 viruses located in the south-east is the most remarkable, consisting of an outbreak in backyard poultry in late November 2021. This was followed by a commercial outbreak in pheasants, identified in late December 2021. However, the load of the A1 virus in the wild bird population must have been quite high in this area, as various A1 strains were detected in swans from early November 2021 to late January 2022.

By contrast, the genotype A2 in wild birds was only known from a single sampling event in early October 2021. This was followed by numerous A2 outbreaks in backyard poultry between November and December 2021, including a critical outbreak in breeding geese. Phylogenetic analysis revealed a clear separation of the strains from geese into a well-supported group with the most recent common ancestor dated to early October 2021 (Supplementary Material file S1, Figure S1). Interestingly, concurrently related strains caused an outbreak in backyard poultry in the region (ID 22224/21). However, the phylogenetic pattern strongly suggests a single introduction event into geese and subsequent secondary spread between farms.

Next, there was a clear genetic link between the genotype C strains, detected in a mute swan in mid-November 2021, and an outbreak in commercial chicken, identified a month apart. On a European scale, genotype C was detected in chickens and turkeys in Italy between November and December 2021 (Supplementary Material file S1, Figure S1). Similarly, genotype D, detected in grey herons between mid-November and December 2021, was similar to the H5N1 strains detected in swans and ducks in Poland between February and March 2022.

Finally, genotypes B, E, and F were not found to have identical genomic constellations in the analyzed dataset. Therefore, they appeared to be unique.

Taken together, the combination of H5 HA and concatenated genome trees suggests two main introductions of the H5N1 HPAI virus in the Czech Republic (genotypes A1 and A2), followed by minor incursions (genotypes B, C, and D). Reassortment of the A2 strains with co-circulating LPAI viruses (including H5N1 LPAI) then led to the genesis of genotypes B, E, and F.

## 4. Discussion

Available data suggests that of the last three European HPAI H5Nx outbreaks, each has surpassed the previous one in severity, in terms of the number of poultry production systems affected, captive and wild birds infected, and the total number of animals culled [1]. In addition, for the first time, Europe experienced two consecutive HPAI seasons with a decrease in avian influenza occurring only during the summer. The transition between seasons is consistent with the observed persistence of H5N1 HPAI in the European wild bird population, when the resident virus pool provided an additional, parallel source for seeding the latest European outbreak [10].

Although the increasing trend in severity between H5Nx outbreak seasons is evident at the European level, at the national level it is not reflected directly. During the last outbreak, France, Italy, and Hungary accounted for ~82% of all cases in poultry, while the sum of H5N1 poultry outbreaks in all other countries was ~18% [1]. Accordingly, the impact of the last HPAI outbreak in the Czech Republic is comparable to that in 2020/2021. Although the number of positive wild birds and affected poultry fluctuated, the overall numbers of birds were roughly identical (*n* = 271,328 during the 2020/2021 season [13] vs. 205,000 in the present study). This was of similar magnitude as was observed during the H5N8 outbreak in 2017 (*n* = 103,144) [14].

As in the previous period, multiple distinct transmissions of the H5N1 HPAI virus from wild birds to domestic poultry populations were observed. Similarly, poultry infections were accompanied by secondary spread between the affected farms. This was probably the most likely scenario in the Pekin duck breeding farms affected in March–April 2021 [13] and also in commercial geese in the current study. However, in the latter case, close evolutionary relationships with backyard poultry in the region suggest more complicated transmission pathways.

Secondary transmission is a frequent way for HPAI to spread between poultry farms in many parts of the EU [35,36,37]. For example, of 750 outbreaks in poultry registered from March–June 2022, 86% were secondary [1] types. Thus, despite the enhanced biosecurity measures implemented throughout the poultry chain, farm-to-farm carryover can be considered the main mechanism of the spread of HPAI in commercial poultry. The cases of secondary transmission observed in the Czech Republic during the 2020/2021 season [13] and also in this study are therefore the rule rather than the exception.

In farmed ducks and geese, HPAI may not cause notifiable clinical signs [38,39,40,41]. In addition, a longer virus shedding period and a higher transmission rate have been observed in ducks [41]. Thus, HPAI in commercial ducks can circulate unrecognized for relatively long periods of time. However, from the onset of flock infection, the virus particles can accumulate to high loads in aerosol [42] and particularly in dust [43]. Both of these fomites are strongly generated in poultry houses. Recent research concluded that contaminated dust particles could be a major mode of viral transmission and dispersal in the environment [43]. Considering that the contaminated dust in poultry houses is omnipresent and adheres to feeds, litter, and other farm facilities, it can also contribute significantly to the secondary transmission of HPAI. In that case, farm-to-farm carryover is indeed unavoidable, even though standard biosecurity measures are in place. Therefore, it seems easier to prevent a primary infection in poultry rather than to avoid a secondary spread. Furthermore, the high viral load in dust suggests the need to re-evaluate current sampling strategies and validate dust sampling for early identification of HPAI in poultry. This appears to be superior to conservative approaches [43].

A remarkable difference between successive HPAI seasons in the Czech Republic is the genotype diversity of the circulated strains. While all Czech H5N8 viruses collected during the 2020/2021 season were genotypically uniform [13], six different H5N1 genotype constellations were found in the following HPAI season. The original A/ew/NL/2020-like genotype was the most widespread, while the others were apparently restricted to a specific location. Three genomes were unique in the dataset analyzed. The observed difference in the genotype spectrum correlates with data at the European level. The avian influenza overview at the end of the 2020/2021 season reports six H5N8 genotypes [8], while 28 H5N1 genotypes have been identified by the end of the 2021/2022 season [1].

We hypothesize that the observed genotype diversity in our data is due to the different timings of the emergence of H5N1 versus H5N8. The first H5N1 strain in 2021 was detected on 27 September, which corresponds to the annual cycle of wild birds and the peak of LPAI virus prevalence in temperate climates [44,45]. This contrasts with the H5N8 HPAI occurrence dated to late January 2021, when the prevalence of co-circulating LPAI viruses is known to be generally low. The emergence of H5N1 HPAI during the peak of LPAI in wild birds provided a great opportunity for the A/ew/NL/2020-like strains to reassort. Moreover, given that all but two (H5 and MP) segments were derived from Eurasian LPAI viruses [5] and the relatively low phenotypic differences between the segments of avian influenza viruses [46], A/ew/NL/2020-like strains are inherently predisposed for great segment exchange with resident LPAI strains.

Accordingly, five distinct phylogenetic lineages of PB2, two of PB1, one of PA, two-to-three of NP, and both alleles of the NS segment were identified among the H5N1 HPAI viruses collected in a relatively small geographic area. This confirms the high propensity of the A/ew/NL/2020-like H5N1 to reassort and implies a whole network of genetic interactions with co-circulating LPAI viruses, specifically H5N1 LPAI. Moreover, given the unique genomic constellation of some Czech H5N1 genotypes, we hypothesize that at least some of them must have arisen relatively shortly before detection. This is suggested by the very close relationship between the NP segment of genotype F (ID 3308/2022) and the co-circulating H5N1 LPAI (ID 20689/21). Another example is the putative second-order relationships between genotypes D and F, which imply additional genomic intermediates.

On the other hand, the genetic diversity found in our data indirectly suggests that the prevalence and diversity of LPAI in the Czech wild bird population during autumn must be considerably high [47]. Unfortunately, this topic is insufficiently studied due to the lack of an active surveillance program. Moreover, genetic distances observed between the Czech H5N1 HPAI segments and the closest LPAI viruses available in the database suggest profound gaps in our knowledge of the circulating LPAI strains in the wild.

The direct transition from one influenza season to another, which occurred from 2020/2021 to 2021/2022, is unprecedented in the recorded history of avian influenza in Europe. For the first time, two parallel outbreak sources were identified, likely representing a side effect of the fundamental change in HPAI infection dynamics in the wild [10]. Both of these phenomena seem to go hand in hand with the global climate changes that we have gradually observed in recent years. This may increase the likelihood of future HPAI outbreaks and present new challenges in poultry management, biosecurity, and surveillance.

## Figures and Tables

**Table 1:** The 2021/2022 H5N1 sampling events listed in chronological order.

Strain ID	Collection Date	Category	Bird Species	Virus Strain and GISAID acc. No.	Genotype
18520	2021-09-27	Backyard poultry	goose	A/goose/Czech_Republic/18520-1/2021|EPI_ISL_5323346	B
			duck	A/duck/Czech_Republic/18520-2/2021|EPI_ISL_5323347	
20689	2021-10-22	Commercial farms	goose	A/goose/Czech_Republic/20689-27T/2021|EPI_ISL_6511912	LPAI
			goose	A/goose/Czech_Republic/20689-28T/2021|EPI_ISL_6512576	
21312	2021-11-01	Wild birds	mute swan	A/mute_swan/Czech_Republic/21312/2021|EPI_ISL_6328409	A1
22224	2021-11-12	Backyard poultry	chicken	A/chicken/Czech_Republic/22224-2T/2021|EPI_ISL_7224437	A2
				A/chicken/Czech_Republic/22224-3K/2021|EPI_ISL_7224454	
				A/chicken/Czech_Republic/22224-3T/2021|EPI_ISL_7224463	
				A/chicken/Czech_Republic/22224-4T/2021|EPI_ISL_7224472	
22380	2021-11-15	Wild birds	mute swan	A/mute_swan/Czech_Republic/22380/2021|EPI_ISL_9603920	C
22477	2021-11-12	Wild birds	mute swan	A/mute_swan/Czech_Republic/22477-1/2021|EPI_ISL_9603916	A1
				A/mute_swan/Czech_Republic/22477-2/2021|EPI_ISL_9603918	
22608 *	2021-11-18	Commercial farms	goose	A/goose/Czech_Republic/22608-1/2021|EPI_ISL_8515478	A2
				A/goose/Czech_Republic/22608-1T/2021|EPI_ISL_9603795	
				A/goose/Czech_Republic/22608-2/2021|EPI_ISL_8515479	
				A/goose/Czech_Republic/22608-2T/2021|EPI_ISL_9603819	
				A/goose/Czech_Republic/22608-3T/2021|EPI_ISL_9603901	
22684	2021-11-15	Wild birds	mute swan	A/mute_swan/Czech_Republic/22684/2021|EPI_ISL_9603922	A1
22750 *	2021-11-19	Commercial farms	goose	A/goose/Czech_Republic/22750/2021|EPI_ISL_9603924	A2
23404	2021-11-25	Backyard poultry	chicken	A/chicken/Czech_Republic/23404-2K/2021|EPI_ISL_7626484	A1
				A/chicken/Czech_Republic/23404-4K/2021|EPI_ISL_7626506	
				A/chicken/Czech_Republic/23404-2T/2021|EPI_ISL_7626512	
				A/chicken/Czech_Republic/23404-4T/2021|EPI_ISL_7626513	
				A/chicken/Czech_Republic/23404/2021|EPI_ISL_8515480	
23458 *	2021-11-26	Commercial farms	goose	A/goose/Czech_Republic/23458-1K/2021|EPI_ISL_9603780	A2
				A/goose/Czech_Republic/23458-2T/2021|EPI_ISL_9603767	
				A/goose/Czech_Republic/23458-4T/2021|EPI_ISL_9603775	
				A/goose/Czech_Republic/23458-5T/2021|EPI_ISL_9603769	
23589	2021-11-29	Backyard poultry	Muscovy duck	A/duck/Czech_Republic/23589-1T/2021|EPI_ISL_7626514	A2
			chicken	A/chicken/Czech_Republic/23589-1T/2021|EPI_ISL_7626526	
				A/chicken/Czech_Republic/23589-3/2021|EPI_ISL_7626532	
				A/chicken/Czech_Republic/23589-4/2021|EPI_ISL_7626533	
23608 $	2021-11-28	Wild birds	grey heron	A/grey heron/Czech_Republic/23608/2021|EPI_ISL_8515481	D
				A/grey heron/Czech_Republic/23608-1K/2021|EPI_ISL8515482	
23609 $	2021-11-28	Wild birds	great egret	A/great egret/Czech_Republic/23609/2021|EPI_ISL_8515483	D
24893 *	2021-12-14	Commercial farms	goose	N.A	N.A
24894 *	2021-12-14	Commercial farms	goose	N.A	N.A
24895 *	2021-12-14	Commercial farms	goose	N.A	N.A
24898 *	2021-12-14	Commercial farms	goose	N.A	N.A
25203	2021-12-16	Backyard poultry	guinea fowl	N.A	N.A
25322 *	2021-12-19	Commercial farms	goose	A/goose/Czech_Republic/25322-179/2021|EPI_ISL_12150664	A2
				A/goose/Czech Republic/25322-229/2021|EPI_ISL_12325995	
				A/goose/Czech_Republic/25322-205/2021|EPI_ISL_12150666	
25324 *	2021-12-19	Commercial farms	goose	N.A	N.A
25338	2021-12-18	Wild birds	grey heron	A/grey_heron/Czech_Republic/25338-1/2021|EPI_ISL_12223688	D
				A/grey_heron/Czech_Republic/25338-2/2021|EPI_ISL_12223734	
25429	2021-12-20	Backyard poultry	chicken	N.A	N.A
25690	2021-12-22	Commercial farms	chicken	A/chicken/Czech_Republic/25690/2021|EPI_ISL_9603929	C
25702	2021-12-20	Wild birds	mute swan	A/mute_swan/Czech_Republic/25702-2/2021|EPI_ISL_9603927	A1
25827	2021-12-25	Commercial farms	pheasant	A/pheasant/Czech_Republic/25827-1/2021|EPI_ISL_9603931	A1
				A/pheasant/Czech_Republic/25827-2/2021|EPI_ISL_9603937	
				A/pheasant/Czech_Republic/25827-3/2021|EPI_ISL_9603939	
61	2021-12-31	Backyard poultry	chicken	A/chicken/Czech_Republic/61-1/2022|EPI_ISL_11327088	A2
				A/chicken/Czech_Republic/61-2/2022|EPI_ISL_11327089	
63	2022-01-03	Backyard poultry	chicken	A/chicken/Czech_Republic/63/2022|EPI_ISL_11327090	A1
65	2022-01-03	Commercial farms	mallard	N.A	N.A
785	2022-01-10	Wild birds	mute swan	A/mute_swan/Czech_Republic/785-/2022|EPI_ISL_12139999	A2
				A/mute_swan/Czech_Republic/785-/2022|EPI_ISL_9603943	
913	2022-01-12	Backyard poultry	chicken	A/chicken/Czech_Republic/913/2022|EPI_ISL_12150658	A2
			goose	A/goose/Czech_Republic/913/2022|EPI_ISL_12150657	
			duck	A/duck/Czech_Republic/913/2022|EPI_ISL_12140437	
1814	2022-01-22	Commercial farms	duck	N.A	N.A
2755	2022-01-29	Wild birds	mute swan	A/mute_swan/Czech_Republic/2755/2022|EPI_ISL_12150661	A1
2968	2022-02-04	Backyard poultry	chicken	HA|A/chicken/Czech_Republic/2968/2022|EPI_ISL_13955171	E
3306	2022-02-09	Backyard poultry	duck	HA|A/duck/Czech_Republic/3306-1/2022|EPI_ISL_12324302	F
				HA|A/chicken/Czech_Republic/3306-2/2022|EPI_ISL_12325210	
4060	2022-02-18	Backyard poultry	chicken	N.A	N.A
4919	2022-03-01	Backyard poultry	N.A	N.A	N.A
8028	2022-04-13	Backyard poultry	chicken	HA|A/chicken/Czech_Republic/8028-1/2022|EPI_ISL_13955204	E
				HA|A/chicken/Czech_Republic/8028-2/2022|EPI_ISL_13967740	

N.A. means not available. * commercial breeding geese from the same company same sampling locality.

## Data Availability

Interactive version of the outbreaks map: https://www.google.com/maps/d/u/0/edit?mid=1_pWrJqfuItU2UwgIuwHjJDsG-tugjU4q&usp=sharing (accessed on 20 November 2022).

## Supplementary Materials

The following supporting information can be downloaded at: https://www.mdpi.com/article/10.3390/v15020293/s1, **Supplementary Material file S1,** Figure S1. Phylogenetic analysis of Czech H5N1 HP/LPAI strains detected during the 2021/2022 influenza season. The ML tree (best-fit substitution models according to Bayesian information criterion: PB2: GTR + F + G4; PB1: UNREST + FO + I + G4; PA: UNREST + FO + G4; H5: GTR + F + G4; NP: GTR + F + I + G4; N1: TVM + F + G4; MP: K3P + G4; NS: TVM + F + I + G4) was calculated separately for each genomic segment, based on Eurasian H5N1 sequences collected between September 2021 and June 2022 and stored in the GISAID EpiFlu database PB2 *n* = 602, PB1 *n* = 591, PA = 593, H5 *n* = 661, NP = 590, N1 *n* = 594, MP *n* = 599 and NS *n* = 597. Coloring: red-commercial, orange-backyard, and blue-wild. For each branch, the bootstrap values (1000 replicates) in percentages are indicated. All trees except N1 were rooted to A/chicken/Scotland/1959 H5N1. The N1 tree was rooted to A/goose/Guangdong/1/1996. The branches of the tree corresponding with discrete genotypes were highlighted with a genotype-specific color (Figure 3). Figure S2. Phylogenetic dating. Concatenated ML tree with the most probable divergence times estimated using the LSD approach. The tree was rooted to the A/ew/NL/1/2020 H5N1 strain. For clarity, only the subtree encompassing genotypes A1 and A2 (Figure 3) was provided. The order of segments in the concatenated genomes is PB2, PB1, PA, H5, NP, N1, MP, and NS. The tree was computed using IQ-TREE (GTR + F + R2 as the best-fitting model selected according to the Bayesian information criterion) and scaled with branch lengths measured in the number of substitutions per site. For each branch, bootstrap values (1000 replicates) were given in percentages. The branches consisting of H5N1 HPAI strains from geese are highlighted in red. Virus taxa were highlighted according to origin: red-commercial; orange-backyard; and blue-wild. **Supplementary Materials file S2.** Sequence identity and sequence difference count matrices of Czech H5N1 HP/LPAI strains detected during the 2021/2022 influenza season. SIMs and SDCMs at nucleic and amino acid levels were calculated from the alignment of coding regions. Data are provided as percentages and absolute values and are visualized as heatmaps. For MP and NS segments, the data were provided for M1, M2, NS1, and NS2/NEP genes, respectively.
