# Supplementary material for: Genotype Diversity, Wild Bird-to-Poultry Transmissions, and Farm-to-Farm Carryover during the Spread of the Highly Pathogenic Avian Influenza H5N1 in the Czech Republic in 2021/2022
Source: Viruses. 2023 Jan 20;15(2):293. doi: 10.3390/v15020293 (PMC9963064; doi:10.3390/v15020293)
Supplement: Supplementary file 1 [file viruses-15-00293-s001.zip › Supplementary Material 1.pdf]

Supplementary Material 1, Table S1. Overview of pathological signs of infected birds. The table shows the frequency of pathological signs in absolute values and in percentages.

Gross pathology lesions	Chicken n=68	Guinea fowl n=3	Duck n=8	Goose n=13	Mute swan n=4	Pheasant n=5	Grey heron/egret n=19
Cyanosis or hyperemia of comb and wattle, head skin or beak	46 (68%)		6 (75%)	9 (69%)			
Cardiac or epicardiac haemorrhages, petechiae or inflammation			3 (38%)	5 (38%)	4 (100%)		2 (11%)
Enlarged spleen	56 (82%)	3 (100%)	6 (75%)	11 (85%)	4 (100%)	5 (100%)	8 (42%)
Serosal haemorrhages	32 (47%)	3 (100%)	7 (88%)	6 (46%)	2 (50%)		4 (21%)
Pancreatic necrosis or haemorrhages	34 (50%)	3 (100%)	6 (75%)	11 (85%)	2 (50%)		8 (42%)
Proventricular haemorrhages or erosions	47 (69%)	3 (100%)	4 (50%)	8 (62%)	2 (50%)		8 (42%)
Lung hyperemia	59 (87%)	3 (100%)	8 (100%)	10 (77%)	4 (100%)	5 (100%)	19 (100%)
Hyperemic trachea	53 (78%)	3 (100%)	7 (88%)	10 (77%)	1 (25%)		8 (42%)
Leg cyanosis or hyperemia	10 (15%)	3 (100%)		5 (38%)			

91-100%
81-90%
71-80 %
61-70 %
51-60%
41-50 %
31-40 %
21-30 %
11-20 %
1-10%

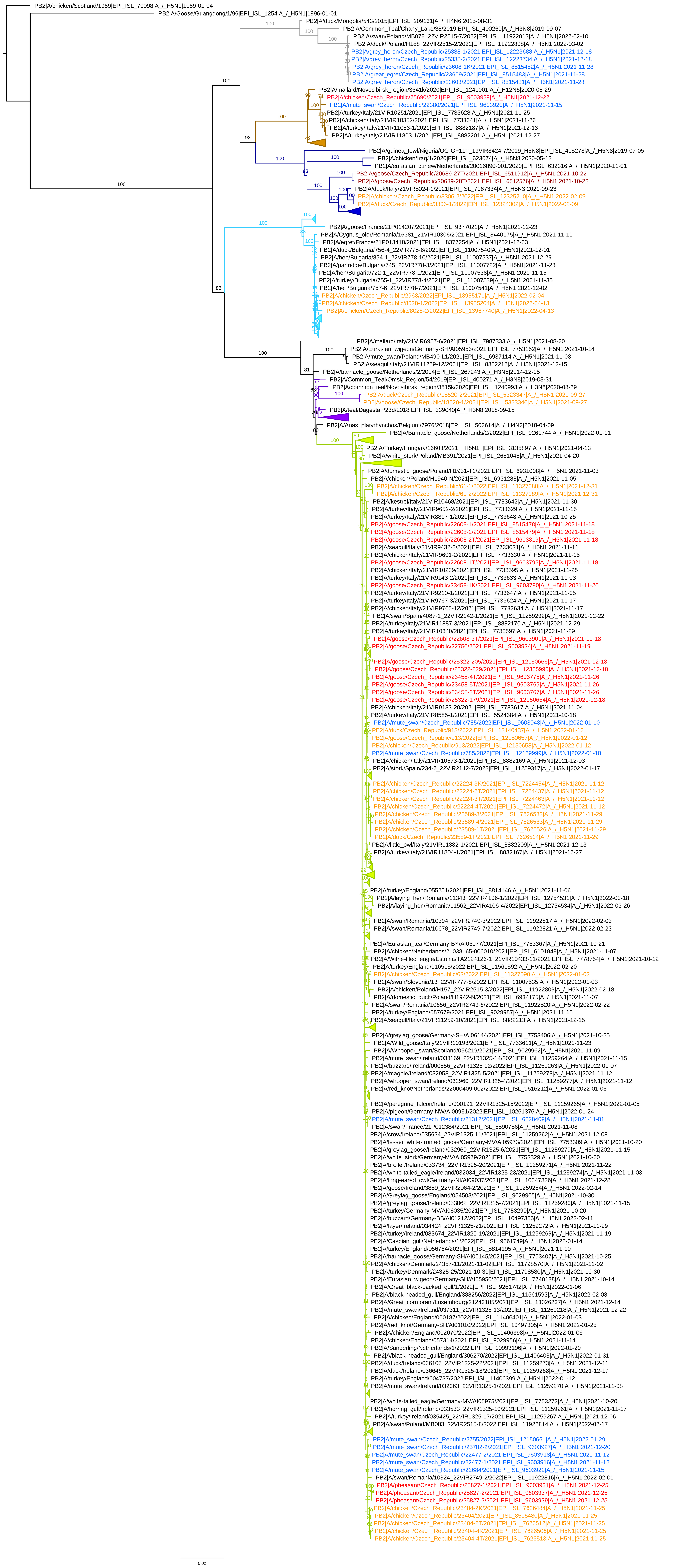
PB2|A/chicken/Scotland/1959|EPI_ISL_70098|A_/_H5N1|1959-01-04
PB2|A/Goose/Guangdong/1/96|EPI_ISL_1254|A_/_H5N1|1996-01-01
PB2|A/duck/Mongolia/543/2015|EPI_ISL_209131|A_/_H4N6|2015-08-31
PB2|A/Common_Teal/Chany_Lake/38/2019|EPI_ISL_400269|A_/_H3N8|2019-09-07
PB2|A/swan/Poland/MB078_22VIR2515-7/2022|EPI_ISL_11922813|A_/_H5N1|2022-02-10
PB2|A/duck/Poland/H188_22VIR2515-2/2022|EPI_ISL_11922808|A_/_H5N1|2022-03-02
PB2|A/grey_heron/Czech_Republic/25338-1/2021|EPI_ISL_12223688|A_/_H5N1|2021-12-18
PB2|A/grey_heron/Czech_Republic/25338-2/2021|EPI_ISL_12223734|A_/_H5N1|2021-12-18
PB2|A/grey_heron/Czech_Republic/23608-1K/2021|EPI_ISL_8515482|A_/_H5N1|2021-11-28
PB2|A/great_egret/Czech_Republic/23609/2021|EPI_ISL_8515483|A_/_H5N1|2021-11-28
PB2|A/grey_heron/Czech_Republic/23608/2021|EPI_ISL_8515481|A_/_H5N1|2021-11-28
PB2|A/mallard/Novosibirsk_region/3541k/2020|EPI_ISL_1241001|A_/_H12N5|2020-08-29
PB2|A/chicken/Czech_Republic/25690/2021|EPI_ISL_9603929|A_/_H5N1|2021-12-22
PB2|A/mute_swan/Czech_Republic/22380/2021|EPI_ISL_9603920|A_/_H5N1|2021-11-15
PB2|A/turkey/Italy/21VIR10251/2021|EPI_ISL_7733628|A_/_H5N1|2021-11-25
PB2|A/chicken/Italy/21VIR10352/2021|EPI_ISL_7733641|A_/_H5N1|2021-11-26
PB2|A/turkey/Italy/21VIR11053-1/2021|EPI_ISL_8882187|A_/_H5N1|2021-12-13
PB2|A/turkey/Italy/21VIR11803-1/2021|EPI_ISL_8882201|A_/_H5N1|2021-12-27
PB2|A/guinea_fowl/Nigeria/OG-GF11T_19VIR8424-7/2019_H5N8|EPI_ISL_405278|A_/_H5N8|2019-07-05
PB2|A/chicken/Iraq/1/2020|EPI_ISL_623074|A_/_H5N8|2020-05-12
PB2|A/eurasian_curlew/Netherlands/20016890-001/2020|EPI_ISL_632316|A_/_H5N1|2020-11-01
PB2|A/goose/Czech_Republic/20689-27T/2021|EPI_ISL_6511912|A_/_H5N1|2021-10-22
PB2|A/goose/Czech_Republic/20689-28T/2021|EPI_ISL_6512576|A_/_H5N1|2021-10-22
PB2|A/duck/Italy/21VIR8024-1/2021|EPI_ISL_7987334|A_/_H5N3|2021-09-23
PB2|A/chicken/Czech_Republic/3306-2/2022|EPI_ISL_12325210|A_/_H5N1|2022-02-09
PB2|A/duck/Czech_Republic/3306-1/2022|EPI_ISL_12324302|A_/_H5N1|2022-02-09
PB2|A/goose/France/21P014207/2021|EPI_ISL_9377021|A_/_H5N1|2021-12-23
PB2|A/Cygnus_olor/Romania/16381_21VIR10306/2021|EPI_ISL_8440175|A_/_H5N1|2021-11-11
PB2|A/egret/France/21P013418/2021|EPI_ISL_8377254|A_/_H5N1|2021-12-03
PB2|A/duck/Bulgaria/756-4_22VIR778-6/2021|EPI_ISL_11007540|A_/_H5N1|2021-12-01
PB2|A/hen/Bulgaria/854-1_22VIR778-10/2021|EPI_ISL_11007537|A_/_H5N1|2021-12-29
PB2|A/partridge/Bulgaria/745_22VIR778-3/2021|EPI_ISL_11007722|A_/_H5N1|2021-11-23
PB2|A/hen/Bulgaria/722-1_22VIR778-1/2021|EPI_ISL_11007538|A_/_H5N1|2021-11-15
PB2|A/turkey/Bulgaria/755-1_22VIR778-4/2021|EPI_ISL_11007539|A_/_H5N1|2021-11-30
PB2|A/hen/Bulgaria/757-6_22VIR778-7/2021|EPI_ISL_11007541|A_/_H5N1|2021-12-02
PB2|A/chicken/Czech_Republic/2968/2022|EPI_ISL_13955171|A_/_H5N1|2022-02-04
PB2|A/chicken/Czech_Republic/8028-1/2022|EPI_ISL_13955204|A_/_H5N1|2022-04-13
PB2|A/chicken/Czech_Republic/8028-2/2022|EPI_ISL_13967740|A_/_H5N1|2022-04-13
PB2|A/mallard/Italy/21VIR6957-6/2021|EPI_ISL_7987333|A_/_H5N1|2021-08-20
PB2|A/Eurasian_wigeon/Germany-SH/AI05953/2021|EPI_ISL_7753152|A_/_H5N1|2021-10-14
PB2|A/mute_swan/Poland/MB490-L1/2021|EPI_ISL_6937114|A_/_H5N1|2021-11-08
PB2|A/seagull/Italy/21VIR11259-12/2021|EPI_ISL_8882218|A_/_H5N1|2021-12-15
PB2|A/barnacle_goose/Netherlands/2/2014|EPI_ISL_267243|A_/_H3N6|2014-12-15
PB2|A/Common_Teal/Omsk_Region/54/2019|EPI_ISL_400271|A_/_H3N8|2019-08-31
PB2|A/common_teal/Novosibirsk_region/3515k/2020|EPI_ISL_1240993|A_/_H3N8|2020-08-29
PB2|A/duck/Czech_Republic/18520-2/2021|EPI_ISL_5323347|A_/_H5N1|2021-09-27
PB2|A/goose/Czech_Republic/18520-1/2021|EPI_ISL_5323346|A_/_H5N1|2021-09-27
PB2|A/teal/Dagestan/23d/2018|EPI_ISL_339040|A_/_H3N8|2018-09-15
PB2|A/Anas_platyrhynchos/Belgium/7976/2018|EPI_ISL_502614|A_/_H4N2|2018-04-09
PB2|A/Barnacle_goose/Netherlands/2/2022|EPI_ISL_9261744|A_/_H5N1|2022-01-11
PB2|A/Turkey/Hungary/16603/2021__H5N1_|EPI_ISL_3135897|A_/_H5N1|2021-04-13
PB2|A/white_stork/Poland/MB391/2021|EPI_ISL_2681045|A_/_H5N1|2021-04-20
PB2|A/domestic_goose/Poland/H1931-T1/2021|EPI_ISL_6931008|A_/_H5N1|2021-11-03
PB2|A/chicken/Poland/H1940-N/2021|EPI_ISL_6931288|A_/_H5N1|2021-11-05
PB2|A/chicken/Czech_Republic/61-1/2022|EPI_ISL_11327088|A_/_H5N1|2021-12-31
PB2|A/chicken/Czech_Republic/61-2/2022|EPI_ISL_11327089|A_/_H5N1|2021-12-31
PB2|A/kestrel/Italy/21VIR10468/2021|EPI_ISL_7733642|A_/_H5N1|2021-11-30
PB2|A/turkey/Italy/21VIR9652-2/2021|EPI_ISL_7733629|A_/_H5N1|2021-11-15
PB2|A/turkey/Italy/21VIR8817-1/2021|EPI_ISL_7733648|A_/_H5N1|2021-10-25
PB2|A/goose/Czech_Republic/22608-1/2021|EPI_ISL_8515478|A_/_H5N1|2021-11-18
PB2|A/goose/Czech_Republic/22608-2/2021|EPI_ISL_8515479|A_/_H5N1|2021-11-18
PB2|A/goose/Czech_Republic/22608-2T/2021|EPI_ISL_9603819|A_/_H5N1|2021-11-18
PB2|A/seagull/Italy/21VIR9432-2/2021|EPI_ISL_7733621|A_/_H5N1|2021-11-11
PB2|A/chicken/Italy/21VIR9691-2/2021|EPI_ISL_7733630|A_/_H5N1|2021-11-15
PB2|A/goose/Czech_Republic/22608-1T/2021|EPI_ISL_9603795|A_/_H5N1|2021-11-18
PB2|A/chicken/Italy/21VIR10239/2021|EPI_ISL_7733595|A_/_H5N1|2021-11-25
PB2|A/turkey/Italy/21VIR9143-2/2021|EPI_ISL_7733633|A_/_H5N1|2021-11-03
PB2|A/goose/Czech_Republic/23458-1K/2021|EPI_ISL_9603780|A_/_H5N1|2021-11-26
PB2|A/turkey/Italy/21VIR9210-1/2021|EPI_ISL_7733647|A_/_H5N1|2021-11-05
PB2|A/turkey/Italy/21VIR9767-3/2021|EPI_ISL_7733624|A_/_H5N1|2021-11-17
PB2|A/chicken/Italy/21VIR9765-12/2021|EPI_ISL_7733634|A_/_H5N1|2021-11-17
PB2|A/swan/Spain/4087-1_22VIR2142-1/2021|EPI_ISL_11259292|A_/_H5N1|2021-12-22
PB2|A/turkey/Italy/21VIR11887-3/2021|EPI_ISL_8882170|A_/_H5N1|2021-12-29
PB2|A/turkey/Italy/21VIR10340/2021|EPI_ISL_7733597|A_/_H5N1|2021-11-29
PB2|A/goose/Czech_Republic/22608-3T/2021|EPI_ISL_9603901|A_/_H5N1|2021-11-18
PB2|A/goose/Czech_Republic/22750/2021|EPI_ISL_9603924|A_/_H5N1|2021-11-19
PB2|A/goose/Czech_Republic/25322-205/2021|EPI_ISL_12150666|A_/_H5N1|2021-12-18
PB2|A/goose/Czech_Republic/25322-229/2021|EPI_ISL_12325995|A_/_H5N1|2021-12-18
PB2|A/goose/Czech_Republic/23458-4T/2021|EPI_ISL_9603775|A_/_H5N1|2021-11-26
PB2|A/goose/Czech_Republic/23458-5T/2021|EPI_ISL_9603769|A_/_H5N1|2021-11-26
PB2|A/goose/Czech_Republic/23458-2T/2021|EPI_ISL_9603767|A_/_H5N1|2021-11-26
PB2|A/goose/Czech_Republic/25322-179/2021|EPI_ISL_12150664|A_/_H5N1|2021-12-18
PB2|A/chicken/Italy/21VIR9133-20/2021|EPI_ISL_7733617|A_/_H5N1|2021-11-04
PB2|A/turkey/Italy/21VIR8585-1/2021|EPI_ISL_5524384|A_/_H5N1|2021-10-18
PB2|A/mute_swan/Czech_Republic/785/2022|EPI_ISL_9603943|A_/_H5N1|2022-01-10
PB2|A/duck/Czech_Republic/913/2022|EPI_ISL_12140437|A_/_H5N1|2022-01-12
PB2|A/goose/Czech_Republic/913/2022|EPI_ISL_12150657|A_/_H5N1|2022-01-12
PB2|A/chicken/Czech_Republic/913/2022|EPI_ISL_12150658|A_/_H5N1|2022-01-12
PB2|A/mute_swan/Czech_Republic/785/2022|EPI_ISL_12139999|A_/_H5N1|2022-01-10
PB2|A/chicken/Italy/21VIR10573-1/2021|EPI_ISL_8882169|A_/_H5N1|2021-12-03
PB2|A/stork/Spain/234-2_22VIR2142-7/2022|EPI_ISL_11259317|A_/_H5N1|2022-01-17
PB2|A/chicken/Czech_Republic/22224-3K/2021|EPI_ISL_7224454|A_/_H5N1|2021-11-12
PB2|A/chicken/Czech_Republic/22224-2T/2021|EPI_ISL_7224437|A_/_H5N1|2021-11-12
PB2|A/chicken/Czech_Republic/22224-3T/2021|EPI_ISL_7224463|A_/_H5N1|2021-11-12
PB2|A/chicken/Czech_Republic/22224-4T/2021|EPI_ISL_7224472|A_/_H5N1|2021-11-12
PB2|A/chicken/Czech_Republic/23589-3/2021|EPI_ISL_7626532|A_/_H5N1|2021-11-29
PB2|A/chicken/Czech_Republic/23589-4/2021|EPI_ISL_7626533|A_/_H5N1|2021-11-29
PB2|A/chicken/Czech_Republic/23589-1T/2021|EPI_ISL_7626526|A_/_H5N1|2021-11-29
PB2|A/duck/Czech_Republic/23589-1T/2021|EPI_ISL_7626514|A_/_H5N1|2021-11-29
PB2|A/little_owl/Italy/21VIR11382-1/2021|EPI_ISL_8882209|A_/_H5N1|2021-12-13
PB2|A/turkey/Italy/21VIR11804-1/2021|EPI_ISL_8882167|A_/_H5N1|2021-12-27
PB2|A/turkey/England/055251/2021|EPI_ISL_8814146|A_/_H5N1|2021-11-06
PB2|A/laying_hen/Romania/11343_22VIR4106-1/2022|EPI_ISL_12754531|A_/_H5N1|2022-03-18
PB2|A/laying_hen/Romania/11562_22VIR4106-4/2022|EPI_ISL_12754534|A_/_H5N1|2022-03-26
PB2|A/swan/Romania/10394_22VIR2749-3/2022|EPI_ISL_11922817|A_/_H5N1|2022-02-03
PB2|A/swan/Romania/10678_22VIR2749-7/2022|EPI_ISL_11922821|A_/_H5N1|2022-02-23
PB2|A/Eurasian_teal/Germany-BY/AI05977/2021|EPI_ISL_7753367|A_/_H5N1|2021-10-21
PB2|A/chicken/Netherlands/21038165-006010/2021|EPI_ISL_6101848|A_/_H5N1|2021-11-07
PB2|A/Withe-tiled_eagle/Estonia/TA2124126-1_21VIR10433-11/2021|EPI_ISL_7778754|A_/_H5N1|2021-10-12
PB2|A/turkey/England/016515/2022|EPI_ISL_11561592|A_/_H5N1|2022-02-20
PB2|A/chicken/Czech_Republic/63/2022|EPI_ISL_11327090|A_/_H5N1|2022-01-03
PB2|A/swan/Slovenia/13_22VIR777-8/2022|EPI_ISL_11007535|A_/_H5N1|2022-01-03
PB2|A/chicken/Poland/H157_22VIR2515-3/2022|EPI_ISL_11922809|A_/_H5N1|2022-02-18
PB2|A/domestic_duck/Poland/H1942-N/2021|EPI_ISL_6934175|A_/_H5N1|2021-11-07
PB2|A/swan/Romania/10656_22VIR2749-6/2022|EPI_ISL_11922820|A_/_H5N1|2022-02-22
PB2|A/turkey/England/057679/2021|EPI_ISL_9029957|A_/_H5N1|2021-11-16
PB2|A/seagull/Italy/21VIR11259-10/2021|EPI_ISL_8882213|A_/_H5N1|2021-12-15
PB2|A/greylag_goose/Germany-SH/AI06144/2021|EPI_ISL_7753406|A_/_H5N1|2021-10-25
PB2|A/Wild_goose/Italy/21VIR10193/2021|EPI_ISL_7733611|A_/_H5N1|2021-11-23
PB2|A/Whooper_swan/Scotland/056219/2021|EPI_ISL_9029962|A_/_H5N1|2021-11-09
PB2|A/mute_swan/Ireland/033169_22VIR1325-14/2021|EPI_ISL_11259264|A_/_H5N1|2021-11-15
PB2|A/buzzard/Ireland/000656_22VIR1325-12/2022|EPI_ISL_11259263|A_/_H5N1|2022-01-07
PB2|A/magpie/Ireland/032958_22VIR1325-5/2021|EPI_ISL_11259278|A_/_H5N1|2021-11-12
PB2|A/whooper_swan/Ireland/032960_22VIR1325-4/2021|EPI_ISL_11259277|A_/_H5N1|2021-11-12
PB2|A/red_knot/Netherlands/22000409-002/2022|EPI_ISL_9616212|A_/_H5N1|2022-01-06
PB2|A/peregrine_falcon/Ireland/000191_22VIR1325-15/2022|EPI_ISL_11259265|A_/_H5N1|2022-01-05
PB2|A/pigeon/Germany-NW/AI00951/2022|EPI_ISL_10261376|A_/_H5N1|2022-01-24
PB2|A/mute_swan/Czech_Republic/21312/2021|EPI_ISL_6328409|A_/_H5N1|2021-11-01
PB2|A/swan/France/21P012384/2021|EPI_ISL_6590766|A_/_H5N1|2021-11-08
PB2|A/crow/Ireland/035624_22VIR1325-11/2021|EPI_ISL_11259262|A_/_H5N1|2021-12-08
PB2|A/lesser_white-fronted_goose/Germany-MV/AI05973/2021|EPI_ISL_7753309|A_/_H5N1|2021-10-20
PB2|A/greylag_goose/Ireland/032969_22VIR1325-6/2021|EPI_ISL_11259279|A_/_H5N1|2021-11-15
PB2|A/white_stork/Germany-MV/AI05979/2021|EPI_ISL_7753329|A_/_H5N1|2021-10-20
PB2|A/broiler/Ireland/033734_22VIR1325-20/2021|EPI_ISL_11259271|A_/_H5N1|2021-11-22
PB2|A/white-tailed_eagle/Ireland/032034_22VIR1325-23/2021|EPI_ISL_11259274|A_/_H5N1|2021-11-03
PB2|A/long-eared_owl/Germany-NI/AI09037/2021|EPI_ISL_10347326|A_/_H5N1|2021-12-28
PB2|A/goose/Ireland/3869_22VIR2064-2/2022|EPI_ISL_11259284|A_/_H5N1|2022-02-14
PB2|A/Greylag_goose/England/054503/2021|EPI_ISL_9029965|A_/_H5N1|2021-10-30
PB2|A/greylag_goose/Ireland/033062_22VIR1325-7/2021|EPI_ISL_11259280|A_/_H5N1|2021-11-15
PB2|A/turkey/Germany-MV/AI06035/2021|EPI_ISL_7753290|A_/_H5N1|2021-10-20
PB2|A/buzzard/Germany-BB/AI01212/2022|EPI_ISL_10497306|A_/_H5N1|2022-02-11
PB2|A/layer/Ireland/034424_22VIR1325-21/2021|EPI_ISL_11259272|A_/_H5N1|2021-11-29
PB2|A/turkey/Ireland/033674_22VIR1325-19/2021|EPI_ISL_11259269|A_/_H5N1|2021-11-19
PB2|A/Caspian_gull/Netherlands/1/2022|EPI_ISL_9261749|A_/_H5N1|2022-01-14
PB2|A/turkey/England/056764/2021|EPI_ISL_8814195|A_/_H5N1|2021-11-10
PB2|A/barnacle_goose/Germany-SH/AI06145/2021|EPI_ISL_7753407|A_/_H5N1|2021-10-25
PB2|A/chicken/Denmark/24357-11/2021-11-02|EPI_ISL_11798570|A_/_H5N1|2021-11-02
PB2|A/turkey/Denmark/24325-25/2021-10-30|EPI_ISL_11798580|A_/_H5N1|2021-10-30
PB2|A/Eurasian_wigeon/Germany-SH/AI05950/2021|EPI_ISL_7748188|A_/_H5N1|2021-10-14
PB2|A/Great_black-backed_gull/1/2022|EPI_ISL_9261742|A_/_H5N1|2022-01-06
PB2|A/black-headed_gull/England/388256/2022|EPI_ISL_11561593|A_/_H5N1|2022-02-03
PB2|A/Great_cormorant/Luxembourg/21243185/2021|EPI_ISL_13026327|A_/_H5N1|2021-12-14
PB2|A/mute_swan/Ireland/037311_22VIR1325-13/2021|EPI_ISL_11260218|A_/_H5N1|2021-12-22
PB2|A/chicken/England/000187/2022|EPI_ISL_11406401|A_/_H5N1|2022-01-03
PB2|A/red_knot/Germany-SH/AI01010/2022|EPI_ISL_10497305|A_/_H5N1|2022-01-25
PB2|A/chicken/England/002070/2022|EPI_ISL_11406398|A_/_H5N1|2022-01-06
PB2|A/chicken/England/057314/2021|EPI_ISL_9029956|A_/_H5N1|2021-11-14
PB2|A/Sanderling/Netherlands/1/2022|EPI_ISL_10993196|A_/_H5N1|2022-01-29
PB2|A/black-headed_gull/England/306270/2022|EPI_ISL_11406403|A_/_H5N1|2022-01-31
PB2|A/duck/Ireland/036105_22VIR1325-22/2021|EPI_ISL_11259273|A_/_H5N1|2021-12-11
PB2|A/duck/Ireland/036646_22VIR1325-18/2021|EPI_ISL_11259268|A_/_H5N1|2021-12-17
PB2|A/turkey/England/004737/2022|EPI_ISL_11406399|A_/_H5N1|2022-01-12
PB2|A/mute_swan/Ireland/032363_22VIR1325-1/2021|EPI_ISL_11259270|A_/_H5N1|2021-11-08
PB2|A/white-tailed_eagle/Germany-MV/AI05975/2021|EPI_ISL_7753272|A_/_H5N1|2021-10-20
PB2|A/herring_gull/Ireland/033533_22VIR1325-10/2021|EPI_ISL_11259261|A_/_H5N1|2021-11-17
PB2|A/turkey/Ireland/035425_22VIR1325-17/2021|EPI_ISL_11259267|A_/_H5N1|2021-12-06
PB2|A/swan/Poland/MB083_22VIR2515-8/2022|EPI_ISL_11922814|A_/_H5N1|2022-02-17
PB2|A/mute_swan/Czech_Republic/2755/2022|EPI_ISL_12150661|A_/_H5N1|2022-01-29
PB2|A/mute_swan/Czech_Republic/25702-1/2021|EPI_ISL_9603927|A_/_H5N1|2021-12-20
PB2|A/mute_swan/Czech_Republic/22477-2/2021|EPI_ISL_9603918|A_/_H5N1|2021-11-12
PB2|A/mute_swan/Czech_Republic/22477-1/2021|EPI_ISL_9603916|A_/_H5N1|2021-11-12
PB2|A/mute_swan/Czech_Republic/22684/2021|EPI_ISL_9603922|A_/_H5N1|2021-11-15
PB2|A/swan/Romania/10324_22VIR2749-2/2022|EPI_ISL_11922816|A_/_H5N1|2022-02-01
PB2|A/pheasant/Czech_Republic/25827-1/2021|EPI_ISL_9603931|A_/_H5N1|2021-12-25
PB2|A/pheasant/Czech_Republic/25827-2/2021|EPI_ISL_9603937|A_/_H5N1|2021-12-25
PB2|A/pheasant/Czech_Republic/25827-3/2021|EPI_ISL_9603939|A_/_H5N1|2021-12-25
PB2|A/chicken/Czech_Republic/23404-2K/2021|EPI_ISL_7626484|A_/_H5N1|2021-11-25
PB2|A/chicken/Czech_Republic/23404/2021|EPI_ISL_8515480|A_/_H5N1|2021-11-25
PB2|A/chicken/Czech_Republic/23404-2T/2021|EPI_ISL_7626512|A_/_H5N1|2021-11-25
PB2|A/chicken/Czech_Republic/23404-4K/2021|EPI_ISL_7626506|A_/_H5N1|2021-11-25
PB2|A/chicken/Czech_Republic/23404-4T/2021|EPI_ISL_7626513|A_/_H5N1|2021-11-25
0.02

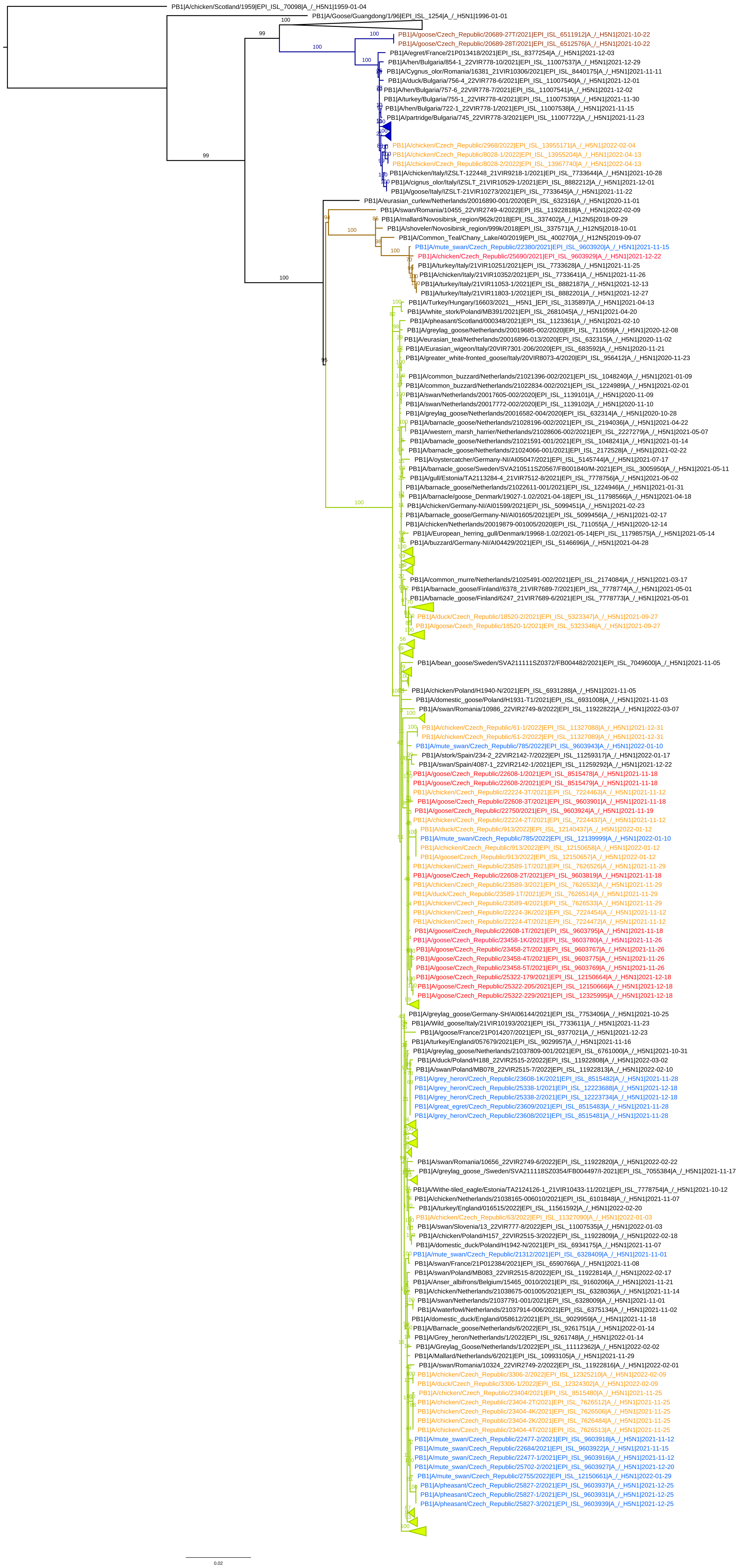
PB1|A/chicken/Scotland/1959|EPI_ISL_70098|A_/_H5N1|1959-01-04
PB1|A/Goose/Guangdong/1/96|EPI_ISL_1254|A_/_H5N1|1996-01-01
PB1|A/goose/Czech_Republic/20689-27T/2021|EPI_ISL_6511912|A_/_H5N1|2021-10-22
PB1|A/goose/Czech_Republic/20689-28T/2021|EPI_ISL_6512576|A_/_H5N1|2021-10-22
PB1|A/egret/France/21P013418/2021|EPI_ISL_8377254|A_/_H5N1|2021-12-03
PB1|A/hen/Bulgaria/854-1_22VIR778-10/2021|EPI_ISL_11007537|A_/_H5N1|2021-12-29
PB1|A/Cygnus_olor/Romania/16381_21VIR10306/2021|EPI_ISL_8440175|A_/_H5N1|2021-11-11
PB1|A/duck/Bulgaria/756-4_22VIR778-6/2021|EPI_ISL_11007540|A_/_H5N1|2021-12-01
PB1|A/hen/Bulgaria/757-6_22VIR778-7/2021|EPI_ISL_11007541|A_/_H5N1|2021-12-02
PB1|A/turkey/Bulgaria/755-1_22VIR778-4/2021|EPI_ISL_11007539|A_/_H5N1|2021-11-30
PB1|A/hen/Bulgaria/722-1_22VIR778-1/2021|EPI_ISL_11007538|A_/_H5N1|2021-11-15
PB1|A/partridge/Bulgaria/745_22VIR778-3/2021|EPI_ISL_11007722|A_/_H5N1|2021-11-23
PB1|A/chicken/Czech_Republic/2968/2022|EPI_ISL_13955171|A_/_H5N1|2022-02-04
PB1|A/chicken/Czech_Republic/8028-1/2022|EPI_ISL_13955204|A_/_H5N1|2022-04-13
PB1|A/chicken/Czech_Republic/8028-2/2022|EPI_ISL_13967740|A_/_H5N1|2022-04-13
PB1|A/chicken/Italy/IZSLT-122448_21VIR9218-1/2021|EPI_ISL_7733644|A_/_H5N1|2021-10-28
PB1|A/cignus_olor/Italy/IZSLT_21VIR10529-1/2021|EPI_ISL_8882212|A_/_H5N1|2021-12-01
PB1|A/goose/Italy/IZSLT-21VIR10273/2021|EPI_ISL_7733645|A_/_H5N1|2021-11-22
PB1|A/eurasian_curlew/Netherlands/20016890-001/2020|EPI_ISL_632316|A_/_H5N1|2020-11-01
PB1|A/swan/Romania/10455_22VIR2749-4/2022|EPI_ISL_11922818|A_/_H5N1|2022-02-09
PB1|A/mallard/Novosibirsk_region/962k/2018|EPI_ISL_337402|A_/_H12N5|2018-09-29
PB1|A/shoveler/Novosibirsk_region/999k/2018|EPI_ISL_337571|A_/_H12N5|2018-10-01
PB1|A/Common_Teal/Chany_Lake/40/2019|EPI_ISL_400270|A_/_H12N5|2019-09-07
PB1|A/mute_swan/Czech_Republic/22380/2021|EPI_ISL_9603920|A_/_H5N1|2021-11-15
PB1|A/chicken/Czech_Republic/25690/2021|EPI_ISL_9603929|A_/_H5N1|2021-12-22
PB1|A/turkey/Italy/21VIR10251/2021|EPI_ISL_7733628|A_/_H5N1|2021-11-25
PB1|A/chicken/Italy/21VIR10352/2021|EPI_ISL_7733641|A_/_H5N1|2021-11-26
PB1|A/turkey/Italy/21VIR11053-1/2021|EPI_ISL_8882187|A_/_H5N1|2021-12-13
PB1|A/turkey/Italy/21VIR11803-1/2021|EPI_ISL_8882201|A_/_H5N1|2021-12-27
PB1|A/Turkey/Hungary/16603/2021__H5N1_|EPI_ISL_3135897|A_/_H5N1|2021-04-13
PB1|A/white_stork/Poland/MB391/2021|EPI_ISL_2681045|A_/_H5N1|2021-04-20
PB1|A/pheasant/Scotland/000348/2021|EPI_ISL_1123361|A_/_H5N1|2021-02-10
PB1|A/greylag_goose/Netherlands/20019685-002/2020|EPI_ISL_711059|A_/_H5N1|2020-12-08
PB1|A/eurasian_teal/Netherlands/20016896-013/2020|EPI_ISL_632315|A_/_H5N1|2020-11-02
PB1|A/Eurasian_wigeon/Italy/20VIR7301-206/2020|EPI_ISL_683592|A_/_H5N1|2020-11-21
PB1|A/greater_white-fronted_goose/Italy/20VIR8073-4/2020|EPI_ISL_956412|A_/_H5N1|2020-11-23
PB1|A/common_buzzard/Netherlands/21021396-002/2021|EPI_ISL_1048240|A_/_H5N1|2021-01-09
PB1|A/common_buzzard/Netherlands/21022834-002/2021|EPI_ISL_1224989|A_/_H5N1|2021-02-01
PB1|A/swan/Netherlands/20017605-002/2020|EPI_ISL_1139101|A_/_H5N1|2020-11-09
PB1|A/swan/Netherlands/20017772-002/2020|EPI_ISL_1139102|A_/_H5N1|2020-11-10
PB1|A/greylag_goose/Netherlands/20016582-004/2020|EPI_ISL_632314|A_/_H5N1|2020-10-28
PB1|A/barnacle_goose/Netherlands/21028196-002/2021|EPI_ISL_2194036|A_/_H5N1|2021-04-22
PB1|A/western_marsh_harrier/Netherlands/21028606-002/2021|EPI_ISL_2227279|A_/_H5N1|2021-05-07
PB1|A/barnacle_goose/Netherlands/21021591-001/2021|EPI_ISL_1048241|A_/_H5N1|2021-01-14
PB1|A/barnacle_goose/Netherlands/21024066-001/2021|EPI_ISL_2172528|A_/_H5N1|2021-02-22
PB1|A/oystercatcher/Germany-NI/AI05047/2021|EPI_ISL_5145744|A_/_H5N1|2021-07-17
PB1|A/barnacle_goose/Sweden/SVA210511SZ0567/FB001840/M-2021|EPI_ISL_3005950|A_/_H5N1|2021-05-11
PB1|A/gull/Estonia/TA2113284-4_21VIR7512-8/2021|EPI_ISL_7778756|A_/_H5N1|2021-06-02
PB1|A/barnacle_goose/Netherlands/21022611-001/2021|EPI_ISL_1224946|A_/_H5N1|2021-01-31
PB1|A/barnacle/goose_Denmark/19027-1.02/2021-04-18|EPI_ISL_11798566|A_/_H5N1|2021-04-18
PB1|A/chicken/Germany-NI/AI01599/2021|EPI_ISL_5099451|A_/_H5N1|2021-02-23
PB1|A/barnacle_goose/Germany-NI/AI01605/2021|EPI_ISL_5099456|A_/_H5N1|2021-02-17
PB1|A/chicken/Netherlands/20019879-001005/2020|EPI_ISL_711055|A_/_H5N1|2020-12-14
PB1|A/European_herring_gull/Denmark/19968-1.02/2021-05-14|EPI_ISL_11798575|A_/_H5N1|2021-05-14
PB1|A/buzzard/Germany-NI/AI04429/2021|EPI_ISL_5146696|A_/_H5N1|2021-04-28
PB1|A/common_murre/Netherlands/21025491-002/2021|EPI_ISL_2174084|A_/_H5N1|2021-03-17
PB1|A/barnacle_goose/Finland//6378_21VIR7689-7/2021|EPI_ISL_7778774|A_/_H5N1|2021-05-01
PB1|A/barnacle_goose/Finland/6247_21VIR7689-6/2021|EPI_ISL_7778773|A_/_H5N1|2021-05-01
PB1|A/duck/Czech_Republic/18520-2/2021|EPI_ISL_5323347|A_/_H5N1|2021-09-27
PB1|A/goose/Czech_Republic/18520-1/2021|EPI_ISL_5323346|A_/_H5N1|2021-09-27
PB1|A/bean_goose/Sweden/SVA211111SZ0372/FB004482/2021|EPI_ISL_7049600|A_/_H5N1|2021-11-05
PB1|A/chicken/Poland/H1940-N/2021|EPI_ISL_6931288|A_/_H5N1|2021-11-05
PB1|A/domestic_goose/Poland/H1931-T1/2021|EPI_ISL_6931008|A_/_H5N1|2021-11-03
PB1|A/swan/Romania/10986_22VIR2749-8/2022|EPI_ISL_11922822|A_/_H5N1|2022-03-07
PB1|A/chicken/Czech_Republic/61-1/2022|EPI_ISL_11327088|A_/_H5N1|2021-12-31
PB1|A/chicken/Czech_Republic/61-2/2022|EPI_ISL_11327089|A_/_H5N1|2021-12-31
PB1|A/mute_swan/Czech_Republic/785/2022|EPI_ISL_9603943|A_/_H5N1|2022-01-10
PB1|A/stork/Spain/234-2_22VIR2142-7/2022|EPI_ISL_11259317|A_/_H5N1|2022-01-17
PB1|A/swan/Spain/4087-1_22VIR2142-1/2021|EPI_ISL_11259292|A_/_H5N1|2021-12-22
PB1|A/goose/Czech_Republic/22608-1/2021|EPI_ISL_8515478|A_/_H5N1|2021-11-18
PB1|A/goose/Czech_Republic/22608-2/2021|EPI_ISL_8515479|A_/_H5N1|2021-11-18
PB1|A/chicken/Czech_Republic/22224-3T/2021|EPI_ISL_7224463|A_/_H5N1|2021-11-12
PB1|A/goose/Czech_Republic/22608-3T/2021|EPI_ISL_9603901|A_/_H5N1|2021-11-18
PB1|A/goose/Czech_Republic/22750/2021|EPI_ISL_9603924|A_/_H5N1|2021-11-19
PB1|A/chicken/Czech_Republic/22224-2T/2021|EPI_ISL_7224437|A_/_H5N1|2021-11-12
PB1|A/duck/Czech_Republic/913/2022|EPI_ISL_12140437|A_/_H5N1|2022-01-12
PB1|A/mute_swan/Czech_Republic/785/2022|EPI_ISL_12139999|A_/_H5N1|2022-01-10
PB1|A/chicken/Czech_Republic/913/2022|EPI_ISL_12150658|A_/_H5N1|2022-01-12
PB1|A/goose/Czech_Republic/913/2022|EPI_ISL_12150657|A_/_H5N1|2022-01-12
PB1|A/chicken/Czech_Republic/23589-1T/2021|EPI_ISL_7626526|A_/_H5N1|2021-11-29
PB1|A/goose/Czech_Republic/22608-2T/2021|EPI_ISL_9603819|A_/_H5N1|2021-11-18
PB1|A/chicken/Czech_Republic/23589-3/2021|EPI_ISL_7626532|A_/_H5N1|2021-11-29
PB1|A/duck/Czech_Republic/23589-1T/2021|EPI_ISL_7626514|A_/_H5N1|2021-11-29
PB1|A/chicken/Czech_Republic/23589-4/2021|EPI_ISL_7626533|A_/_H5N1|2021-11-29
PB1|A/chicken/Czech_Republic/22224-3K/2021|EPI_ISL_7224454|A_/_H5N1|2021-11-12
PB1|A/chicken/Czech_Republic/22224-4T/2021|EPI_ISL_7224472|A_/_H5N1|2021-11-12
PB1|A/goose/Czech_Republic/22608-1T/2021|EPI_ISL_9603795|A_/_H5N1|2021-11-18
PB1|A/goose/Czech_Republic/23458-1K/2021|EPI_ISL_9603780|A_/_H5N1|2021-11-26
PB1|A/goose/Czech_Republic/23458-2T/2021|EPI_ISL_9603767|A_/_H5N1|2021-11-26
PB1|A/goose/Czech_Republic/23458-4T/2021|EPI_ISL_9603775|A_/_H5N1|2021-11-26
PB1|A/goose/Czech_Republic/23458-5T/2021|EPI_ISL_9603769|A_/_H5N1|2021-11-26
PB1|A/goose/Czech_Republic/25322-179/2021|EPI_ISL_12150664|A_/_H5N1|2021-12-18
PB1|A/goose/Czech_Republic/25322-205/2021|EPI_ISL_12150666|A_/_H5N1|2021-12-18
PB1|A/goose/Czech_Republic/25322-229/2021|EPI_ISL_12325995|A_/_H5N1|2021-12-18
PB1|A/greylag_goose/Germany-SH/AI06144/2021|EPI_ISL_7753406|A_/_H5N1|2021-10-25
PB1|A/Wild_goose/Italy/21VIR10193/2021|EPI_ISL_7733611|A_/_H5N1|2021-11-23
PB1|A/goose/France/21P014207/2021|EPI_ISL_9377021|A_/_H5N1|2021-12-23
PB1|A/turkey/England/057679/2021|EPI_ISL_9029957|A_/_H5N1|2021-11-16
PB1|A/greylag_goose/Netherlands/21037809-001/2021|EPI_ISL_6761000|A_/_H5N1|2021-10-31
PB1|A/duck/Poland/H188_22VIR2515-2/2022|EPI_ISL_11922808|A_/_H5N1|2022-03-02
PB1|A/swan/Poland/MB078_22VIR2515-7/2022|EPI_ISL_11922813|A_/_H5N1|2022-02-10
PB1|A/grey_heron/Czech_Republic/23608-1K/2021|EPI_ISL_8515482|A_/_H5N1|2021-11-28
PB1|A/grey_heron/Czech_Republic/25338-1/2021|EPI_ISL_12223688|A_/_H5N1|2021-12-18
PB1|A/grey_heron/Czech_Republic/25338-2/2021|EPI_ISL_12223734|A_/_H5N1|2021-12-18
PB1|A/great_egret/Czech_Republic/23609/2021|EPI_ISL_8515483|A_/_H5N1|2021-11-28
PB1|A/grey_heron/Czech_Republic/23608/2021|EPI_ISL_8515481|A_/_H5N1|2021-11-28
PB1|A/swan/Romania/10656_22VIR2749-6/2022|EPI_ISL_11922820|A_/_H5N1|2022-02-22
PB1|A/greylag_goose_/Sweden/SVA211118SZ0354/FB004497/I-2021|EPI_ISL_7055384|A_/_H5N1|2021-11-17
PB1|A/Withe-tiled_eagle/Estonia/TA2124126-1_21VIR10433-11/2021|EPI_ISL_7778754|A_/_H5N1|2021-10-12
PB1|A/chicken/Netherlands/21038165-006010/2021|EPI_ISL_6101848|A_/_H5N1|2021-11-07
PB1|A/turkey/England/016515/2022|EPI_ISL_11561592|A_/_H5N1|2022-02-20
PB1|A/chicken/Czech_Republic/63/2022|EPI_ISL_11327090|A_/_H5N1|2022-01-03
PB1|A/swan/Slovenia/13_22VIR777-8/2022|EPI_ISL_11007535|A_/_H5N1|2022-01-03
PB1|A/chicken/Poland/H157_22VIR2515-3/2022|EPI_ISL_11922809|A_/_H5N1|2022-02-18
PB1|A/domestic_duck/Poland/H1942-N/2021|EPI_ISL_6934175|A_/_H5N1|2021-11-07
PB1|A/mute_swan/Czech_Republic/21312/2021|EPI_ISL_6328409|A_/_H5N1|2021-11-01
PB1|A/swan/France/21P012384/2021|EPI_ISL_6590766|A_/_H5N1|2021-11-08
PB1|A/swan/Poland/MB083_22VIR2515-8/2022|EPI_ISL_11922814|A_/_H5N1|2022-02-17
PB1|A/Anser_albifrons/Belgium/15465_0010/2021|EPI_ISL_9160206|A_/_H5N1|2021-11-21
PB1|A/chicken/Netherlands/21038675-001005/2021|EPI_ISL_6328036|A_/_H5N1|2021-11-14
PB1|A/swan/Netherlands/21037791-001/2021|EPI_ISL_6328009|A_/_H5N1|2021-11-01
PB1|A/waterfowl/Netherlands/21037914-006/2021|EPI_ISL_6375134|A_/_H5N1|2021-11-02
PB1|A/domestic_duck/England/058612/2021|EPI_ISL_9029959|A_/_H5N1|2021-11-18
PB1|A/Barnacle_goose/Netherlands/6/2022|EPI_ISL_9261751|A_/_H5N1|2022-01-14
PB1|A/Grey_heron/Netherlands/1/2022|EPI_ISL_9261748|A_/_H5N1|2022-01-14
PB1|A/Greylag_Goose/Netherlands/1/2022|EPI_ISL_11112362|A_/_H5N1|2022-02-02
PB1|A/Mallard/Netherlands/6/2021|EPI_ISL_10993105|A_/_H5N1|2021-11-29
PB1|A/swan/Romania/10324_22VIR2749-2/2022|EPI_ISL_11922816|A_/_H5N1|2022-02-01
PB1|A/chicken/Czech_Republic/3306-2/2022|EPI_ISL_12325210|A_/_H5N1|2022-02-09
PB1|A/duck/Czech_Republic/3306-1/2022|EPI_ISL_12324302|A_/_H5N1|2022-02-09
PB1|A/chicken/Czech_Republic/23404/2021|EPI_ISL_8515480|A_/_H5N1|2021-11-25
PB1|A/chicken/Czech_Republic/23404-2T/2021|EPI_ISL_7626512|A_/_H5N1|2021-11-25
PB1|A/chicken/Czech_Republic/23404-4K/2021|EPI_ISL_7626506|A_/_H5N1|2021-11-25
PB1|A/chicken/Czech_Republic/23404-2K/2021|EPI_ISL_7626484|A_/_H5N1|2021-11-25
PB1|A/chicken/Czech_Republic/23404-4T/2021|EPI_ISL_7626513|A_/_H5N1|2021-11-25
PB1|A/mute_swan/Czech_Republic/22477-2/2021|EPI_ISL_9603918|A_/_H5N1|2021-11-12
PB1|A/mute_swan/Czech_Republic/22684/2021|EPI_ISL_9603922|A_/_H5N1|2021-11-15
PB1|A/mute_swan/Czech_Republic/22477-1/2021|EPI_ISL_9603916|A_/_H5N1|2021-11-12
PB1|A/mute_swan/Czech_Republic/25702-2/2021|EPI_ISL_9603927|A_/_H5N1|2021-12-20
PB1|A/mute_swan/Czech_Republic/2755/2022|EPI_ISL_12150661|A_/_H5N1|2022-01-29
PB1|A/pheasant/Czech_Republic/25827-2/2021|EPI_ISL_9603937|A_/_H5N1|2021-12-25
PB1|A/pheasant/Czech_Republic/25827-1/2021|EPI_ISL_9603931|A_/_H5N1|2021-12-25
PB1|A/pheasant/Czech_Republic/25827-3/2021|EPI_ISL_9603939|A_/_H5N1|2021-12-25
0.02

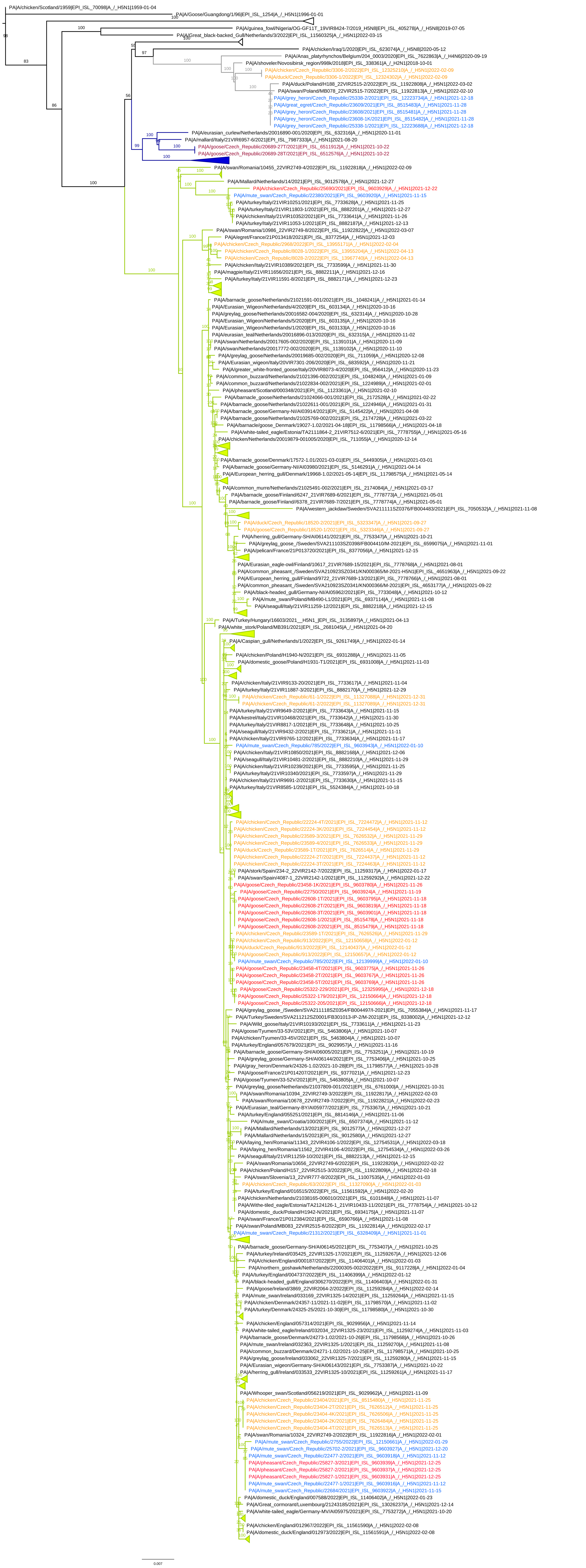

PA|A/chicken/Scotland/1959|EPI_ISL_70098|A_/_H5N1|1959-01-04
PA|A/Goose/Guangdong/1/96|EPI_ISL_1254|A_/_H5N1|1996-01-01
PA|A/guinea_fowl/Nigeria/OG-GF11T_19VIR8424-7/2019_H5N8|EPI_ISL_405278|A_/_H5N8|2019-07-05
PA|A/Great_black-backed_Gull/Netherlands/3/2022|EPI_ISL_11560325|A_/_H5N1|2022-03-15
PA|A/chicken/Iraq/1/2020|EPI_ISL_623074|A_/_H5N8|2020-05-12
PA|A/Anas_platyrhynchos/Belgium/204_0003/2020|EPI_ISL_7622863|A_/_H4N6|2020-09-19
PA|A/shoveler/Novosibirsk_region/998k/2018|EPI_ISL_338361|A_/_H2N1|2018-10-01
PA|A/chicken/Czech_Republic/3306-2/2022|EPI_ISL_12325210|A_/_H5N1|2022-02-09
PA|A/duck/Czech_Republic/3306-1/2022|EPI_ISL_12324302|A_/_H5N1|2022-02-09
PA|A/duck/Poland/H188_22VIR2515-2/2022|EPI_ISL_11922808|A_/_H5N1|2022-03-02
PA|A/swan/Poland/MB078_22VIR2515-7/2022|EPI_ISL_11922813|A_/_H5N1|2022-02-10
PA|A/grey_heron/Czech_Republic/25338-2/2021|EPI_ISL_12223734|A_/_H5N1|2021-12-18
PA|A/great_egret/Czech_Republic/23609/2021|EPI_ISL_8515483|A_/_H5N1|2021-11-28
PA|A/grey_heron/Czech_Republic/23608/2021|EPI_ISL_8515481|A_/_H5N1|2021-11-28
PA|A/grey_heron/Czech_Republic/23608-1K/2021|EPI_ISL_8515482|A_/_H5N1|2021-11-28
PA|A/grey_heron/Czech_Republic/25338-1/2021|EPI_ISL_12223688|A_/_H5N1|2021-12-18
PA|A/eurasian_curlew/Netherlands/20016890-001/2020|EPI_ISL_632316|A_/_H5N1|2020-11-01
PA|A/mallard/Italy/21VIR6957-6/2021|EPI_ISL_7987333|A_/_H5N1|2021-08-20
PA|A/goose/Czech_Republic/20689-27T/2021|EPI_ISL_6511912|A_/_H5N1|2021-10-22
PA|A/goose/Czech_Republic/20689-28T/2021|EPI_ISL_6512576|A_/_H5N1|2021-10-22
PA|A/swan/Romania/10455_22VIR2749-4/2022|EPI_ISL_11922818|A_/_H5N1|2022-02-09
PA|A/Mallard/Netherlands/14/2021|EPI_ISL_9012578|A_/_H5N1|2021-12-27
PA|A/chicken/Czech_Republic/25690/2021|EPI_ISL_9603929|A_/_H5N1|2021-12-22
PA|A/mute_swan/Czech_Republic/22380/2021|EPI_ISL_9603920|A_/_H5N1|2021-11-15
PA|A/turkey/Italy/21VIR10251/2021|EPI_ISL_7733628|A_/_H5N1|2021-11-25
PA|A/turkey/Italy/21VIR11803-1/2021|EPI_ISL_8882201|A_/_H5N1|2021-12-27
PA|A/chicken/Italy/21VIR10352/2021|EPI_ISL_7733641|A_/_H5N1|2021-11-26
PA|A/turkey/Italy/21VIR11053-1/2021|EPI_ISL_8882187|A_/_H5N1|2021-12-13
PA|A/swan/Romania/10986_22VIR2749-8/2022|EPI_ISL_11922822|A_/_H5N1|2022-03-07
PA|A/egret/France/21P013418/2021|EPI_ISL_8377254|A_/_H5N1|2021-12-03
PA|A/chicken/Czech_Republic/2968/2022|EPI_ISL_13955171|A_/_H5N1|2022-02-04
PA|A/chicken/Czech_Republic/8028-1/2022|EPI_ISL_13955204|A_/_H5N1|2022-04-13
PA|A/chicken/Czech_Republic/8028-2/2022|EPI_ISL_13967740|A_/_H5N1|2022-04-13
PA|A/chicken/Italy/21VIR10389/2021|EPI_ISL_7733599|A_/_H5N1|2021-11-30
PA|A/magpie/Italy/21VIR11656/2021|EPI_ISL_8882211|A_/_H5N1|2021-12-16
PA|A/turkey/Italy/21VIR11591-8/2021|EPI_ISL_8882171|A_/_H5N1|2021-12-23
PA|A/barnacle_goose/Netherlands/21021591-001/2021|EPI_ISL_1048241|A_/_H5N1|2021-01-14
PA|A/Eurasian_Wigeon/Netherlands/4/2020|EPI_ISL_603134|A_/_H5N1|2020-10-16
PA|A/greylag_goose/Netherlands/20016582-004/2020|EPI_ISL_632314|A_/_H5N1|2020-10-28
PA|A/Eurasian_Wigeon/Netherlands/5/2020|EPI_ISL_603135|A_/_H5N1|2020-10-16
PA|A/Eurasian_Wigeon/Netherlands/1/2020|EPI_ISL_603133|A_/_H5N1|2020-10-16
PA|A/eurasian_teal/Netherlands/20016896-013/2020|EPI_ISL_632315|A_/_H5N1|2020-11-02
PA|A/swan/Netherlands/20017605-002/2020|EPI_ISL_1139101|A_/_H5N1|2020-11-09
PA|A/swan/Netherlands/20017772-002/2020|EPI_ISL_1139102|A_/_H5N1|2020-11-10
PA|A/greylag_goose/Netherlands/20019685-002/2020|EPI_ISL_711059|A_/_H5N1|2020-12-08
PA|A/Eurasian_wigeon/Italy/20VIR7301-206/2020|EPI_ISL_683592|A_/_H5N1|2020-11-21
PA|A/greater_white-fronted_goose/Italy/20VIR8073-4/2020|EPI_ISL_956412|A_/_H5N1|2020-11-23
PA|A/common_buzzard/Netherlands/21021396-002/2021|EPI_ISL_1048240|A_/_H5N1|2021-01-09
PA|A/common_buzzard/Netherlands/21022834-002/2021|EPI_ISL_1224989|A_/_H5N1|2021-02-01
PA|A/pheasant/Scotland/000348/2021|EPI_ISL_1123361|A_/_H5N1|2021-02-10
PA|A/barnacle_goose/Netherlands/21024066-001/2021|EPI_ISL_2172528|A_/_H5N1|2021-02-22
PA|A/barnacle_goose/Netherlands/21022611-001/2021|EPI_ISL_1224946|A_/_H5N1|2021-01-31
PA|A/barnacle_goose/Germany-NI/AI03914/2021|EPI_ISL_5145422|A_/_H5N1|2021-04-08
PA|A/barnacle_goose/Netherlands/21025769-002/2021|EPI_ISL_2174728|A_/_H5N1|2021-03-22
PA|A/barnacle/goose_Denmark/19027-1.02/2021-04-18|EPI_ISL_11798566|A_/_H5N1|2021-04-18
PA|A/white-tailed_eagle/Estonia/TA2111864-2_21VIR7512-6/2021|EPI_ISL_7778755|A_/_H5N1|2021-05-16
PA|A/chicken/Netherlands/20019879-001005/2020|EPI_ISL_711055|A_/_H5N1|2020-12-14
PA|A/barnacle_goose/Denmark/17572-1.01/2021-03-01|EPI_ISL_5449305|A_/_H5N1|2021-03-01
PA|A/barnacle_goose/Germany-NI/AI03980/2021|EPI_ISL_5146291|A_/_H5N1|2021-04-14
PA|A/European_herring_gull/Denmark/19968-1.02/2021-05-14|EPI_ISL_11798575|A_/_H5N1|2021-05-14
PA|A/common_murre/Netherlands/21025491-002/2021|EPI_ISL_2174084|A_/_H5N1|2021-03-17
PA|A/barnacle_goose/Finland/6247_21VIR7689-6/2021|EPI_ISL_7778773|A_/_H5N1|2021-05-01
PA|A/barnacle_goose/Finland//6378_21VIR7689-7/2021|EPI_ISL_7778774|A_/_H5N1|2021-05-01
PA|A/western_jackdaw/Sweden/SVA211111SZ0376/FB004483/2021|EPI_ISL_7050532|A_/_H5N1|2021-11-08
PA|A/duck/Czech_Republic/18520-2/2021|EPI_ISL_5323347|A_/_H5N1|2021-09-27
PA|A/chicken/Czech_Republic/18520-1/2021|EPI_ISL_5323346|A_/_H5N1|2021-09-27
PA|A/herring_gull/Germany-SH/AI06141/2021|EPI_ISL_7753347|A_/_H5N1|2021-10-21
PA|A/greylag_goose_/Sweden/SVA211103SZ0398/FB004410/M-2021|EPI_ISL_6599075|A_/_H5N1|2021-11-01
PA|A/pelican/France/21P013720/2021|EPI_ISL_8377056|A_/_H5N1|2021-12-15
PA|A/Eurasian_eagle-owl/Finland/10617_21VIR7689-15/2021|EPI_ISL_7778768|A_/_H5N1|2021-08-01
PA|A/common_pheasant_/Sweden/SVA210923SZ0341/KN000365/M-2021-H5N1|EPI_ISL_4651963|A_/_H5N1|2021-09-22
PA|A/European_herring_gull/Finland/9722_21VIR7689-13/2021|EPI_ISL_7778766|A_/_H5N1|2021-08-01
PA|A/common_pheasant_/Sweden/SVA210923SZ0341/KN000366/M-2021|EPI_ISL_4653177|A_/_H5N1|2021-09-22
PA|A/black-headed_gull/Germany-NI/AI05962/2021|EPI_ISL_7733048|A_/_H5N1|2021-10-12
PA|A/mute_swan/Poland/MB490-L1/2021|EPI_ISL_6937114|A_/_H5N1|2021-11-08
PA|A/seagull/Italy/21VIR11259-12/2021|EPI_ISL_8882218|A_/_H5N1|2021-12-15
PA|A/Turkey/Hungary/16603/2021__H5N1_|EPI_ISL_3135897|A_/_H5N1|2021-04-13
PA|A/white_stork/Poland/MB391/2021|EPI_ISL_2681045|A_/_H5N1|2021-04-20
PA|A/Caspian_gull/Netherlands/1/2022|EPI_ISL_9261749|A_/_H5N1|2022-01-14
PA|A/chicken/Poland/H1940-N/2021|EPI_ISL_6931288|A_/_H5N1|2021-11-05
PA|A/domestic_goose/Poland/H1931-T1/2021|EPI_ISL_6931008|A_/_H5N1|2021-11-03
PA|A/chicken/Italy/21VIR9133-20/2021|EPI_ISL_7733617|A_/_H5N1|2021-11-04
PA|A/turkey/Italy/21VIR11887-3/2021|EPI_ISL_8882170|A_/_H5N1|2021-12-29
PA|A/chicken/Czech_Republic/61-1/2022|EPI_ISL_11327088|A_/_H5N1|2021-12-31
PA|A/chicken/Czech_Republic/61-2/2022|EPI_ISL_11327089|A_/_H5N1|2021-12-31
PA|A/turkey/Italy/21VIR9649-2/2021|EPI_ISL_7733643|A_/_H5N1|2021-11-15
PA|A/kestrel/Italy/21VIR10468/2021|EPI_ISL_7733642|A_/_H5N1|2021-11-30
PA|A/turkey/Italy/21VIR8817-1/2021|EPI_ISL_7733648|A_/_H5N1|2021-10-25
PA|A/seagull/Italy/21VIR9432-2/2021|EPI_ISL_7733621|A_/_H5N1|2021-11-11
PA|A/chicken/Italy/21VIR9765-12/2021|EPI_ISL_7733634|A_/_H5N1|2021-11-17
PA|A/mute_swan/Czech_Republic/785/2022|EPI_ISL_9603943|A_/_H5N1|2022-01-10
PA|A/chicken/Italy/21VIR10850/2021|EPI_ISL_8882168|A_/_H5N1|2021-12-06
PA|A/seagull/Italy/21VIR10481-2/2021|EPI_ISL_8882210|A_/_H5N1|2021-11-29
PA|A/chicken/Italy/21VIR10239/2021|EPI_ISL_7733595|A_/_H5N1|2021-11-25
PA|A/turkey/Italy/21VIR10340/2021|EPI_ISL_7733597|A_/_H5N1|2021-11-29
PA|A/chicken/Italy/21VIR9691-2/2021|EPI_ISL_7733630|A_/_H5N1|2021-11-15
PA|A/turkey/Italy/21VIR8585-1/2021|EPI_ISL_5524384|A_/_H5N1|2021-10-18
PA|A/chicken/Czech_Republic/22224-4T/2021|EPI_ISL_7224472|A_/_H5N1|2021-11-12
PA|A/chicken/Czech_Republic/22224-3K/2021|EPI_ISL_7224454|A_/_H5N1|2021-11-12
PA|A/chicken/Czech_Republic/23589-3/2021|EPI_ISL_7626532|A_/_H5N1|2021-11-29
PA|A/chicken/Czech_Republic/23589-4/2021|EPI_ISL_7626533|A_/_H5N1|2021-11-29
PA|A/duck/Czech_Republic/23589-1T/2021|EPI_ISL_7626514|A_/_H5N1|2021-11-29
PA|A/chicken/Czech_Republic/22224-2T/2021|EPI_ISL_7224437|A_/_H5N1|2021-11-12
PA|A/chicken/Czech_Republic/22224-3T/2021|EPI_ISL_7224463|A_/_H5N1|2021-11-12
PA|A/stork/Spain/234-2_22VIR2142-7/2022|EPI_ISL_11259317|A_/_H5N1|2022-01-17
PA|A/swan/Spain/4087-1_22VIR2142-1/2021|EPI_ISL_11259292|A_/_H5N1|2021-12-22
PA|A/goose/Czech_Republic/23458-1K/2021|EPI_ISL_9603780|A_/_H5N1|2021-11-26
PA|A/goose/Czech_Republic/22750/2021|EPI_ISL_9603924|A_/_H5N1|2021-11-19
PA|A/goose/Czech_Republic/22608-1T/2021|EPI_ISL_9603795|A_/_H5N1|2021-11-18
PA|A/goose/Czech_Republic/22608-2T/2021|EPI_ISL_9603819|A_/_H5N1|2021-11-18
PA|A/goose/Czech_Republic/22608-3T/2021|EPI_ISL_9603901|A_/_H5N1|2021-11-18
PA|A/goose/Czech_Republic/22608-1/2021|EPI_ISL_8515478|A_/_H5N1|2021-11-18
PA|A/goose/Czech_Republic/22608-2/2021|EPI_ISL_8515479|A_/_H5N1|2021-11-18
PA|A/chicken/Czech_Republic/23589-1T/2021|EPI_ISL_7626526|A_/_H5N1|2021-11-29
PA|A/chicken/Czech_Republic/913/2022|EPI_ISL_12150658|A_/_H5N1|2022-01-12
PA|A/duck/Czech_Republic/913/2022|EPI_ISL_12140437|A_/_H5N1|2022-01-12
PA|A/goose/Czech_Republic/913/2022|EPI_ISL_12150657|A_/_H5N1|2022-01-12
PA|A/mute_swan/Czech_Republic/785/2022|EPI_ISL_12139999|A_/_H5N1|2022-01-10
PA|A/goose/Czech_Republic/23458-4T/2021|EPI_ISL_9603775|A_/_H5N1|2021-11-26
PA|A/goose/Czech_Republic/23458-2T/2021|EPI_ISL_9603767|A_/_H5N1|2021-11-26
PA|A/goose/Czech_Republic/23458-5T/2021|EPI_ISL_9603769|A_/_H5N1|2021-11-26
PA|A/goose/Czech_Republic/25322-229/2021|EPI_ISL_12325995|A_/_H5N1|2021-12-18
PA|A/goose/Czech_Republic/25322-179/2021|EPI_ISL_12150664|A_/_H5N1|2021-12-18
PA|A/goose/Czech_Republic/25322-205/2021|EPI_ISL_12150666|A_/_H5N1|2021-12-18
PA|A/greylag_goose_/Sweden/SVA211118SZ0354/FB004497/I-2021|EPI_ISL_7055384|A_/_H5N1|2021-11-17
PA|A/Turkey/Sweden/SVA211212SZ0001/FB301013-IP-2/M-2021|EPI_ISL_8338002|A_/_H5N1|2021-12-12
PA|A/Wild_goose/Italy/21VIR10193/2021|EPI_ISL_7733611|A_/_H5N1|2021-11-23
PA|A/goose/Tyumen/33-53V/2021|EPI_ISL_5463806|A_/_H5N1|2021-10-07
PA|A/chicken/Tyumen/33-45V/2021|EPI_ISL_5463804|A_/_H5N1|2021-10-07
PA|A/turkey/England/057679/2021|EPI_ISL_9029957|A_/_H5N1|2021-11-16
PA|A/barnacle_goose/Germany-SH/AI06005/2021|EPI_ISL_7753251|A_/_H5N1|2021-10-19
PA|A/greylag_goose/Germany-SH/AI06144/2021|EPI_ISL_7753406|A_/_H5N1|2021-10-25
PA|A/gray_heron/Denmark/24326-1.02/2021-10-28|EPI_ISL_11798577|A_/_H5N1|2021-10-28
PA|A/goose/France/21P014207/2021|EPI_ISL_9377021|A_/_H5N1|2021-12-23
PA|A/goose/Tyumen/33-52V/2021|EPI_ISL_5463805|A_/_H5N1|2021-10-07
PA|A/greylag_goose/Netherlands/21037809-001/2021|EPI_ISL_6761000|A_/_H5N1|2021-10-31
PA|A/swan/Romania/10394_22VIR2749-3/2022|EPI_ISL_11922817|A_/_H5N1|2022-02-03
PA|A/swan/Romania/10678_22VIR2749-7/2022|EPI_ISL_11922821|A_/_H5N1|2022-02-23
PA|A/Eurasian_teal/Germany-BR/AI05977/2021|EPI_ISL_7753367|A_/_H5N1|2021-10-21
PA|A/turkey/England/055251/2021|EPI_ISL_8814146|A_/_H5N1|2021-11-06
PA|A/mute_swan/Croatia/100/2021|EPI_ISL_6507374|A_/_H5N1|2021-11-12
PA|A/Mallard/Netherlands/13/2021|EPI_ISL_9012577|A_/_H5N1|2021-12-27
PA|A/Mallard/Netherlands/15/2021|EPI_ISL_9012580|A_/_H5N1|2021-12-27
PA|A/laying_hen/Romania/11343_22VIR4106-1/2022|EPI_ISL_12754531|A_/_H5N1|2022-03-18
PA|A/laying_hen/Romania/11562_22VIR4106-4/2022|EPI_ISL_12754534|A_/_H5N1|2022-03-26
PA|A/seagull/Italy/21VIR11259-10/2021|EPI_ISL_8882213|A_/_H5N1|2021-12-15
PA|A/swan/Romania/10656_22VIR2749-6/2022|EPI_ISL_11922820|A_/_H5N1|2022-02-22
PA|A/chicken/Poland/H157_22VIR2515-3/2022|EPI_ISL_11922809|A_/_H5N1|2022-02-18
PA|A/swan/Slovenia/13_22VIR777-8/2022|EPI_ISL_11007535|A_/_H5N1|2022-01-03
PA|A/chicken/Czech_Republic/63/2022|EPI_ISL_11327090|A_/_H5N1|2022-01-03
PA|A/turkey/England/016515/2022|EPI_ISL_11561592|A_/_H5N1|2022-02-20
PA|A/chicken/Netherlands/21038165-006010/2021|EPI_ISL_6101848|A_/_H5N1|2021-11-07
PA|A/Withe-tiled_eagle/Estonia/TA2124126-1_21VIR10433-11/2021|EPI_ISL_7778754|A_/_H5N1|2021-10-12
PA|A/domestic_duck/Poland/H1942-N/2021|EPI_ISL_6934175|A_/_H5N1|2021-11-07
PA|A/swan/France/21P012384/2021|EPI_ISL_6590766|A_/_H5N1|2021-11-08
PA|A/swan/Poland/MB083_22VIR2515-8/2022|EPI_ISL_11922814|A_/_H5N1|2022-02-17
PA|A/mute_swan/Czech_Republic/21312/2021|EPI_ISL_6328409|A_/_H5N1|2021-11-01
PA|A/barnacle_goose/Germany-SH/AI06145/2021|EPI_ISL_7753407|A_/_H5N1|2021-10-25
PA|A/turkey/Ireland/035425_22VIR1325-17/2021|EPI_ISL_11259267|A_/_H5N1|2021-12-06
PA|A/chicken/England/000187/2022|EPI_ISL_11406401|A_/_H5N1|2022-01-03
PA|A/northern_goshawk/Netherlands/22000305-002/2022|EPI_ISL_9117228|A_/_H5N1|2022-01-04
PA|A/turkey/England/004737/2022|EPI_ISL_11406399|A_/_H5N1|2022-01-12
PA|A/black-headed_gull/England/306270/2022|EPI_ISL_11406403|A_/_H5N1|2022-01-31
PA|A/goose/Ireland/3869_22VIR2064-2/2022|EPI_ISL_11259284|A_/_H5N1|2022-02-14
PA|A/mute_swan/Ireland/033169_22VIR1325-14/2021|EPI_ISL_11259264|A_/_H5N1|2021-11-15
PA|A/chicken/Denmark/24357-11/2021-11-02|EPI_ISL_11798570|A_/_H5N1|2021-11-02
PA|A/turkey/Denmark/24325-25/2021-10-30|EPI_ISL_11798580|A_/_H5N1|2021-10-30
PA|A/chicken/England/057314/2021|EPI_ISL_9029956|A_/_H5N1|2021-11-14
PA|A/white-tailed_eagle/Ireland/032034_22VIR1325-23/2021|EPI_ISL_11259274|A_/_H5N1|2021-11-03
PA|A/barnacle_goose/Denmark/24273-1.02/2021-10-26|EPI_ISL_11798568|A_/_H5N1|2021-10-26
PA|A/mute_swan/Ireland/032363_22VIR1325-1/2021|EPI_ISL_11259270|A_/_H5N1|2021-11-08
PA|A/common_buzzard/Denmark/24271-1.02/2021-10-25|EPI_ISL_11798571|A_/_H5N1|2021-10-25
PA|A/greylag_goose/Ireland/033062_22VIR1325-7/2021|EPI_ISL_11259280|A_/_H5N1|2021-11-15
PA|A/Eurasian_wigeon/Germany-SH/AI06143/2021|EPI_ISL_7753387|A_/_H5N1|2021-10-22
PA|A/herring_gull/Ireland/033533_22VIR1325-10/2021|EPI_ISL_11259261|A_/_H5N1|2021-11-17
PA|A/Whooper_swan/Scotland/056219/2021|EPI_ISL_9029962|A_/_H5N1|2021-11-09
PA|A/chicken/Czech_Republic/23404/2021|EPI_ISL_8515480|A_/_H5N1|2021-11-25
PA|A/chicken/Czech_Republic/23404-2T/2021|EPI_ISL_7626512|A_/_H5N1|2021-11-25
PA|A/chicken/Czech_Republic/23404-4K/2021|EPI_ISL_7626506|A_/_H5N1|2021-11-25
PA|A/chicken/Czech_Republic/23404-2K/2021|EPI_ISL_7626484|A_/_H5N1|2021-11-25
PA|A/chicken/Czech_Republic/23404-4T/2021|EPI_ISL_7626513|A_/_H5N1|2021-11-25
PA|A/swan/Romania/10324_22VIR2749-2/2022|EPI_ISL_11922816|A_/_H5N1|2022-02-01
PA|A/mute_swan/Czech_Republic/2755/2022|EPI_ISL_12150661|A_/_H5N1|2022-01-29
PA|A/mute_swan/Czech_Republic/25702-2/2021|EPI_ISL_9603927|A_/_H5N1|2021-12-20
PA|A/mute_swan/Czech_Republic/22477-2/2021|EPI_ISL_9603918|A_/_H5N1|2021-11-12
PA|A/pheasant/Czech_Republic/25827-3/2021|EPI_ISL_9603939|A_/_H5N1|2021-12-25
PA|A/pheasant/Czech_Republic/25827-2/2021|EPI_ISL_9603937|A_/_H5N1|2021-12-25
PA|A/pheasant/Czech_Republic/25827-1/2021|EPI_ISL_9603931|A_/_H5N1|2021-12-25
PA|A/mute_swan/Czech_Republic/22477-1/2021|EPI_ISL_9603916|A_/_H5N1|2021-11-12
PA|A/mute_swan/Czech_Republic/22684/2021|EPI_ISL_9603922|A_/_H5N1|2021-11-15
PA|A/domestic_duck/England/007588/2022|EPI_ISL_11406402|A_/_H5N1|2022-01-23
PA|A/Great_cormorant/Luxembourg/21243185/2021|EPI_ISL_13026237|A_/_H5N1|2021-12-14
PA|A/white-tailed_eagle/Germany-MV/AI05975/2021|EPI_ISL_7753272|A_/_H5N1|2021-10-20
PA|A/chicken/England/012967/2022|EPI_ISL_11561590|A_/_H5N1|2022-02-08
PA|A/domestic_duck/England/012973/2022|EPI_ISL_11561591|A_/_H5N1|2022-02-08
0.007

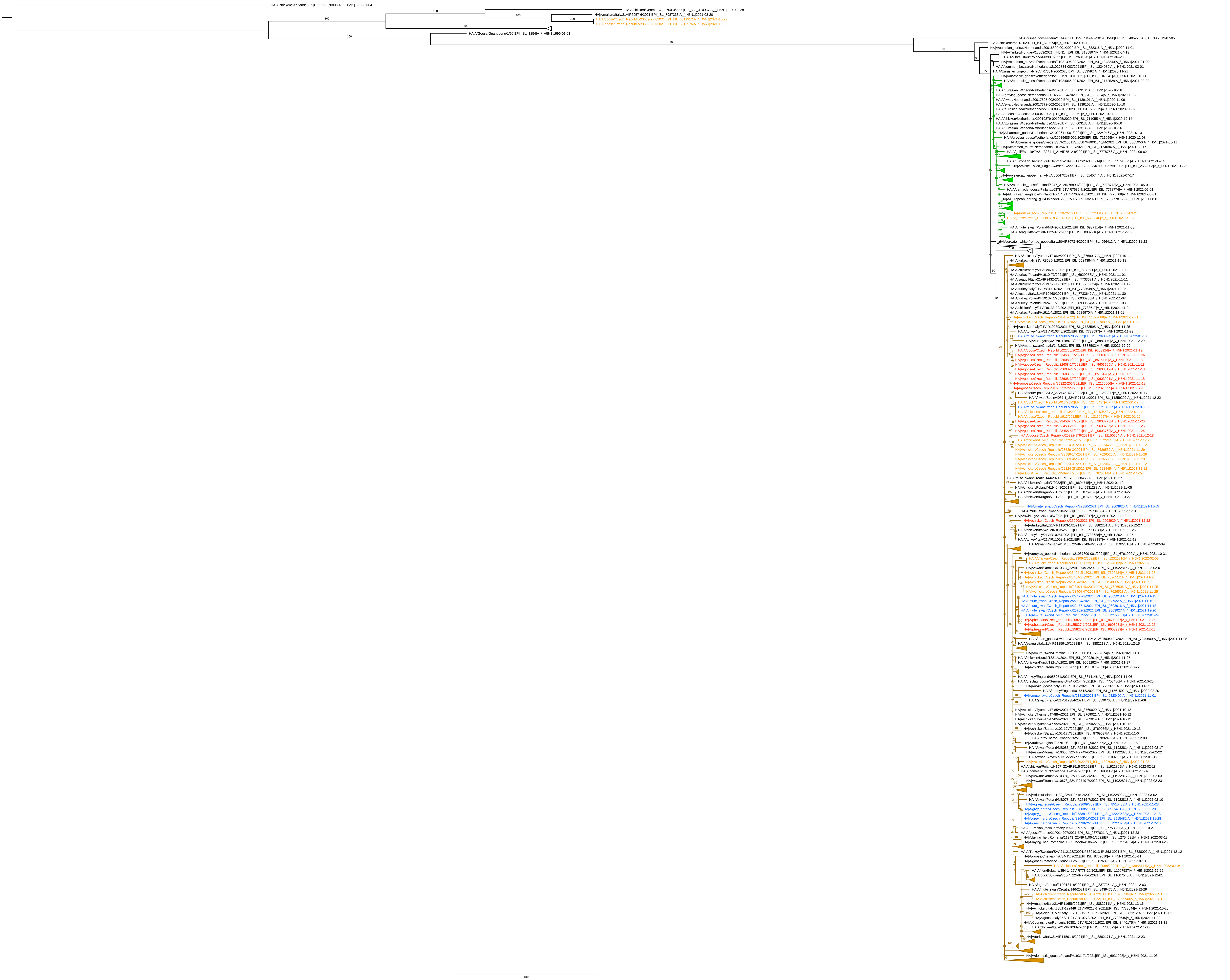
HA|A/chicken/Scotland/1959|EPI_ISL_70098|A_/_H5N1|1959-01-04
HA|A/chicken/Denmark/S02750-3/2020|EPI_ISL_410987|A_/_H5N1|2020-01-28
HA|A/mallard/Italy/21VIR6957-6/2021|EPI_ISL_7987333|A_/_H5N1|2021-08-20
HA|A/Goose/Guangdong/1/96|EPI_ISL_1254|A_/_H5N1|1996-01-01
HA|A/guinea_fowl/Nigeria/OG-GF11T_19VIR8424-7/2019_H5N8|EPI_ISL_405278|A_/_H5N8|2019-07-05
0.03

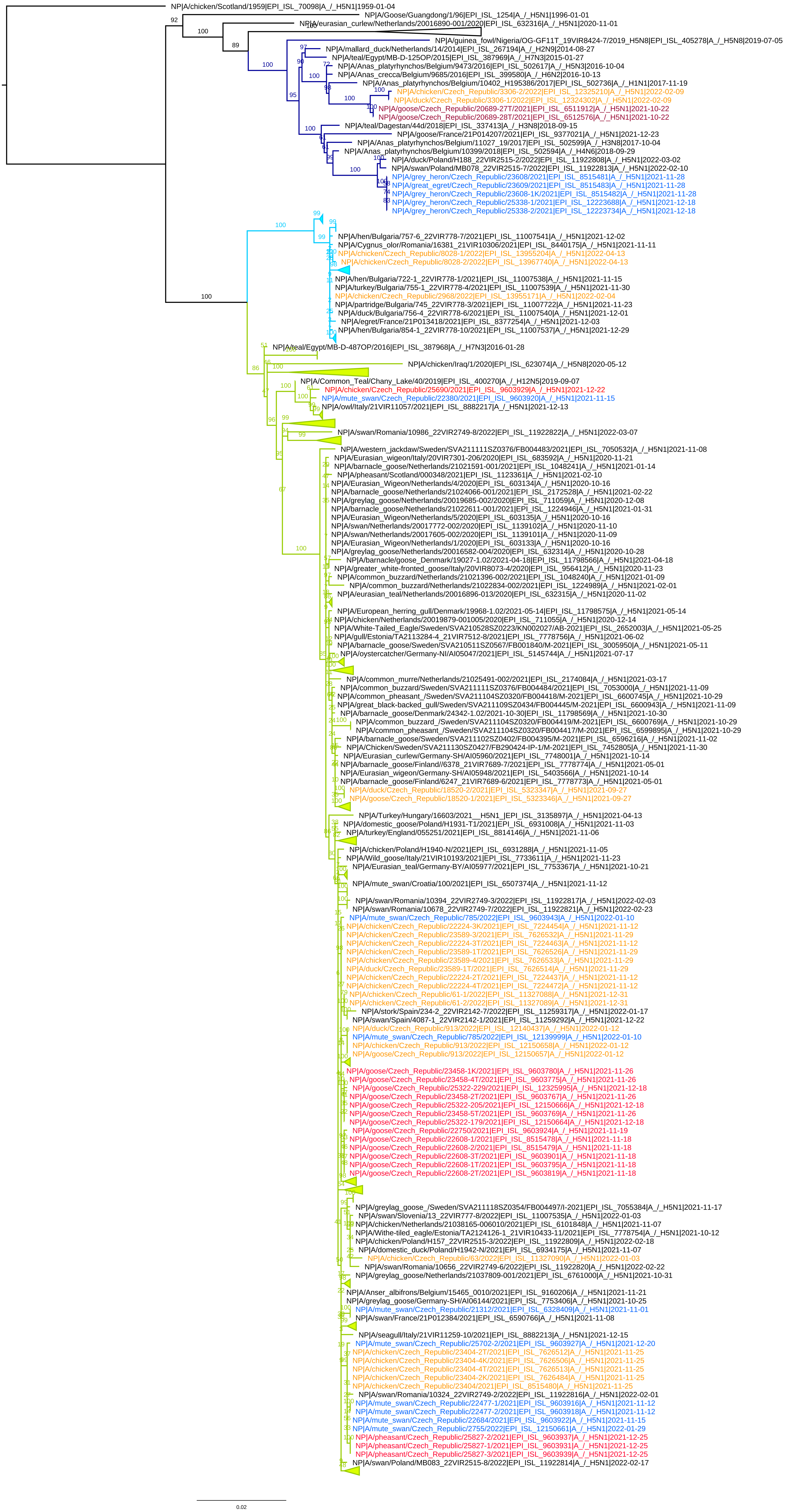
NP|A/chicken/Scotland/1959|EPI_ISL_70098|A_/_H5N1|1959-01-04
NP|A/Goose/Guangdong/1/96|EPI_ISL_1254|A_/_H5N1|1996-01-01
NP|A/eurasian_curlew/Netherlands/20016890-001/2020|EPI_ISL_632316|A_/_H5N1|2020-11-01
NP|A/guinea_fowl/Nigeria/OG-GF11T_19VIR8424-7/2019_H5N8|EPI_ISL_405278|A_/_H5N8|2019-07-05
NP|A/mallard_duck/Netherlands/14/2014|EPI_ISL_267196|A_/_H2N9|2014-08-27
NP|A/teal/Egypt/MB-D-125OP/2015|EPI_ISL_387969|A_/_H7N3|2015-01-27
NP|A/Anas_platyrhynchos/Belgium/9473/2016|EPI_ISL_502617|A_/_H5N3|2016-10-04
NP|A/Anas_crecca/Belgium/9685/2016|EPI_ISL_399580|A_/_H6N2|2016-10-13
NP|A/Anas_platyrhynchos/Belgium/10402_H195386/2017|EPI_ISL_502736|A_/_H1N1|2017-11-19
NP|A/chicken/Czech_Republic/3306-2/2022|EPI_ISL_12325210|A_/_H5N1|2022-02-09
NP|A/duck/Czech_Republic/3306-1/2022|EPI_ISL_12324302|A_/_H5N1|2022-02-09
NP|A/goose/Czech_Republic/20689-27T/2021|EPI_ISL_6511912|A_/_H5N1|2021-10-22
NP|A/goose/Czech_Republic/20689-28T/2021|EPI_ISL_6512576|A_/_H5N1|2021-10-22
NP|A/teal/Dagestan/44d/2018|EPI_ISL_337413|A_/_H3N8|2018-09-15
NP|A/goose/France/21P014207/2021|EPI_ISL_9377021|A_/_H5N1|2021-12-23
NP|A/Anas_platyrhynchos/Belgium/11027_19/2017|EPI_ISL_502599|A_/_H3N8|2017-10-04
NP|A/Anas_platyrhynchos/Belgium/10399/2018|EPI_ISL_502594|A_/_H4N6|2018-09-29
NP|A/duck/Poland/H188_22VIR2515-2/2022|EPI_ISL_11922808|A_/_H5N1|2022-03-02
NP|A/swan/Poland/MB078_22VIR2515-7/2022|EPI_ISL_11922813|A_/_H5N1|2022-02-10
NP|A/grey_heron/Czech_Republic/23608/2021|EPI_ISL_8515481|A_/_H5N1|2021-11-28
NP|A/great_egret/Czech_Republic/23609/2021|EPI_ISL_8515483|A_/_H5N1|2021-11-28
NP|A/grey_heron/Czech_Republic/23608-1K/2021|EPI_ISL_8515482|A_/_H5N1|2021-11-28
NP|A/grey_heron/Czech_Republic/25338-1/2021|EPI_ISL_12223688|A_/_H5N1|2021-12-18
NP|A/grey_heron/Czech_Republic/25338-2/2021|EPI_ISL_12223734|A_/_H5N1|2021-12-18
NP|A/hen/Bulgaria/757-6_22VIR778-7/2021|EPI_ISL_11007541|A_/_H5N1|2021-12-02
NP|A/Cygnus_olor/Romania/16381_21VIR10306/2021|EPI_ISL_8440175|A_/_H5N1|2021-11-11
NP|A/chicken/Czech_Republic/8028-1/2022|EPI_ISL_13955204|A_/_H5N1|2022-04-13
NP|A/chicken/Czech_Republic/8028-2/2022|EPI_ISL_13967740|A_/_H5N1|2022-04-13
NP|A/hen/Bulgaria/722-1_22VIR778-1/2021|EPI_ISL_11007538|A_/_H5N1|2021-11-15
NP|A/turkey/Bulgaria/755-1_22VIR778-4/2021|EPI_ISL_11007539|A_/_H5N1|2021-11-30
NP|A/chicken/Czech_Republic/2968/2022|EPI_ISL_13955171|A_/_H5N1|2022-04-04
NP|A/partridge/Bulgaria/745_22VIR778-3/2021|EPI_ISL_11007722|A_/_H5N1|2021-11-23
NP|A/duck/Bulgaria/756-4_22VIR778-6/2021|EPI_ISL_11007540|A_/_H5N1|2021-12-01
NP|A/egret/France/21P013418/2021|EPI_ISL_8377254|A_/_H5N1|2021-12-03
NP|A/hen/Bulgaria/854-1_22VIR778-10/2021|EPI_ISL_11007537|A_/_H5N1|2021-12-29
NP|A/teal/Egypt/MB-D-487OP/2016|EPI_ISL_387968|A_/_H7N3|2016-01-28
NP|A/chicken/Iraq/1/2020|EPI_ISL_623074|A_/_H5N8|2020-05-12
NP|A/Common_Teal/Chany_Lake/40/2019|EPI_ISL_400270|A_/_H12N5|2019-09-07
NP|A/chicken/Czech_Republic/25690/2021|EPI_ISL_9603929|A_/_H5N1|2021-12-22
NP|A/mute_swan/Czech_Republic/22380/2021|EPI_ISL_9603920|A_/_H5N1|2021-11-15
NP|A/owl/Italy/21VIR11057/2021|EPI_ISL_8882217|A_/_H5N1|2021-12-13
NP|A/swan/Romania/10986_22VIR2749-8/2022|EPI_ISL_11922822|A_/_H5N1|2022-03-07
NP|A/western_jackdaw/Sweden/SVA211111SZ0376/FB004483/2021|EPI_ISL_7050532|A_/_H5N1|2021-11-08
NP|A/Eurasian_wigeon/Italy/20VIR7301-206/2020|EPI_ISL_683592|A_/_H5N1|2020-11-21
NP|A/barnacle_goose/Netherlands/21021591-001/2021|EPI_ISL_1048241|A_/_H5N1|2021-01-14
NP|A/pheasant/Scotland/000348/2021|EPI_ISL_1123361|A_/_H5N1|2021-02-10
NP|A/Eurasian_Wigeon/Netherlands/4/2020|EPI_ISL_603134|A_/_H5N1|2020-10-16
NP|A/barnacle_goose/Netherlands/21024066-001/2021|EPI_ISL_2172528|A_/_H5N1|2021-02-22
NP|A/greylag_goose/Netherlands/20019685-002/2020|EPI_ISL_711059|A_/_H5N1|2020-12-08
NP|A/barnacle_goose/Netherlands/21022611-001/2021|EPI_ISL_1224946|A_/_H5N1|2021-01-31
NP|A/Eurasian_Wigeon/Netherlands/5/2020|EPI_ISL_603135|A_/_H5N1|2020-10-16
NP|A/swan/Netherlands/20017772-002/2020|EPI_ISL_1139102|A_/_H5N1|2020-11-10
NP|A/swan/Netherlands/20017605-002/2020|EPI_ISL_1139101|A_/_H5N1|2020-11-09
NP|A/Eurasian_Wigeon/Netherlands/1/2020|EPI_ISL_603133|A_/_H5N1|2020-10-16
NP|A/greylag_goose/Netherlands/20016582-004/2020|EPI_ISL_632314|A_/_H5N1|2020-10-28
NP|A/barnacle/goose_Denmark/19027-1.02/2021-04-18|EPI_ISL_11798566|A_/_H5N1|2021-04-18
NP|A/greater_white-fronted_goose/Italy/20VIR8073-4/2020|EPI_ISL_956412|A_/_H5N1|2020-11-23
NP|A/common_buzzard/Netherlands/21021396-002/2021|EPI_ISL_1048240|A_/_H5N1|2021-01-09
NP|A/common_buzzard/Netherlands/21022834-002/2021|EPI_ISL_1224989|A_/_H5N1|2021-02-01
NP|A/eurasian_teal/Netherlands/20016896-013/2020|EPI_ISL_632315|A_/_H5N1|2020-11-02
NP|A/European_herring_gull/Denmark/19968-1.02/2021-05-14|EPI_ISL_11798575|A_/_H5N1|2021-05-14
NP|A/chicken/Netherlands/20019879-001005/2020|EPI_ISL_711055|A_/_H5N1|2020-12-14
NP|A/White-Tailed_Eagle/Sweden/SVA210528SZ0223/KN002027/AB-2021|EPI_ISL_2652003|A_/_H5N1|2021-05-25
NP|A/gull/Estonia/TA2113284-4_21VIR7512-8/2021|EPI_ISL_7778756|A_/_H5N1|2021-06-02
NP|A/barnacle_goose/Sweden/SVA210511SZ0567/FB001840/M-2021|EPI_ISL_3005950|A_/_H5N1|2021-05-11
NP|A/oystercatcher/Germany-NI/AI05047/2021|EPI_ISL_5145744|A_/_H5N1|2021-07-17
NP|A/common_murre/Netherlands/21025491-002/2021|EPI_ISL_2174084|A_/_H5N1|2021-03-17
NP|A/common_buzzard/Sweden/SVA211111SZ0376/FB004484/2021|EPI_ISL_7053000|A_/_H5N1|2021-11-09
NP|A/common_pheasant_/Sweden/SVA211104SZ0320/FB004418/M-2021|EPI_ISL_6600745|A_/_H5N1|2021-10-29
NP|A/great_black-backed_gull/Sweden/SVA211109SZ0434/FB004445/M-2021|EPI_ISL_6600943|A_/_H5N1|2021-11-09
NP|A/barnacle_goose/Denmark/24342-1.02/2021-10-30|EPI_ISL_11798569|A_/_H5N1|2021-10-30
NP|A/common_buzzard_/Sweden/SVA211104SZ0320/FB004419/M-2021|EPI_ISL_6600769|A_/_H5N1|2021-10-29
NP|A/common_pheasant_/Sweden/SVA211104SZ0320/FB004417/M-2021|EPI_ISL_6599895|A_/_H5N1|2021-10-29
NP|A/barnacle_goose/Sweden/SVA211102SZ0402/FB004395/M-2021|EPI_ISL_6596216|A_/_H5N1|2021-11-02
NP|A/Chicken/Sweden/SVA211130SZ0427/FB290424-IP-1/M-2021|EPI_ISL_7452805|A_/_H5N1|2021-11-30
NP|A/Eurasian_curlew/Germany-SH/AI05960/2021|EPI_ISL_7748001|A_/_H5N1|2021-10-14
NP|A/barnacle_goose/Finland//6378_21VIR7689-7/2021|EPI_ISL_7778774|A_/_H5N1|2021-05-01
NP|A/Eurasian_wigeon/Germany-SH/AI05948/2021|EPI_ISL_5403566|A_/_H5N1|2021-10-14
NP|A/barnacle_goose/Finland/6247_21VIR7689-6/2021|EPI_ISL_7778773|A_/_H5N1|2021-05-01
NP|A/duck/Czech_Republic/18520-2/2021|EPI_ISL_5323347|A_/_H5N1|2021-09-27
NP|A/goose/Czech_Republic/18520-1/2021|EPI_ISL_5323346|A_/_H5N1|2021-09-27
NP|A/Turkey/Hungary/16603/2021__H5N1_|EPI_ISL_3135897|A_/_H5N1|2021-04-13
NP|A/domestic_goose/Poland/H1931-T1/2021|EPI_ISL_6931008|A_/_H5N1|2021-11-03
NP|A/turkey/England/055251/2021|EPI_ISL_8814146|A_/_H5N1|2021-11-06
NP|A/chicken/Poland/H1940-N/2021|EPI_ISL_6931288|A_/_H5N1|2021-11-05
NP|A/Wild_goose/Italy/21VIR10193/2021|EPI_ISL_7733611|A_/_H5N1|2021-11-23
NP|A/Eurasian_teal/Germany-BY/AI05977/2021|EPI_ISL_7753367|A_/_H5N1|2021-10-21
NP|A/mute_swan/Croatia/100/2021|EPI_ISL_6507374|A_/_H5N1|2021-11-12
NP|A/swan/Romania/10394_22VIR2749-3/2022|EPI_ISL_11922817|A_/_H5N1|2022-02-03
NP|A/swan/Romania/10678_22VIR2749-7/2022|EPI_ISL_11922821|A_/_H5N1|2022-02-23
NP|A/mute_swan/Czech_Republic/785/2022|EPI_ISL_9603943|A_/_H5N1|2022-01-10
NP|A/chicken/Czech_Republic/22224-3K/2021|EPI_ISL_7224454|A_/_H5N1|2021-11-12
NP|A/chicken/Czech_Republic/23589-3/2021|EPI_ISL_7626532|A_/_H5N1|2021-11-29
NP|A/chicken/Czech_Republic/22224-3T/2021|EPI_ISL_7224463|A_/_H5N1|2021-11-12
NP|A/chicken/Czech_Republic/23589-1T/2021|EPI_ISL_7626526|A_/_H5N1|2021-11-29
NP|A/chicken/Czech_Republic/23589-4/2021|EPI_ISL_7626533|A_/_H5N1|2021-11-29
NP|A/duck/Czech_Republic/23589-1T/2021|EPI_ISL_7626514|A_/_H5N1|2021-11-29
NP|A/chicken/Czech_Republic/22224-2T/2021|EPI_ISL_7224437|A_/_H5N1|2021-11-12
NP|A/chicken/Czech_Republic/22224-4T/2021|EPI_ISL_7224472|A_/_H5N1|2021-11-12
NP|A/chicken/Czech_Republic/61-1/2022|EPI_ISL_11327088|A_/_H5N1|2021-12-31
NP|A/chicken/Czech_Republic/61-2/2022|EPI_ISL_11327089|A_/_H5N1|2021-12-31
NP|A/stork/Spain/234-2_22VIR2142-7/2022|EPI_ISL_11259317|A_/_H5N1|2022-01-17
NP|A/swan/Spain/4087-1_22VIR2142-1/2021|EPI_ISL_11259292|A_/_H5N1|2021-12-22
NP|A/duck/Czech_Republic/913/2022|EPI_ISL_12140437|A_/_H5N1|2022-01-12
NP|A/mute_swan/Czech_Republic/785/2022|EPI_ISL_12139999|A_/_H5N1|2022-01-10
NP|A/chicken/Czech_Republic/913/2022|EPI_ISL_12150658|A_/_H5N1|2022-01-12
NP|A/goose/Czech_Republic/913/2022|EPI_ISL_12150657|A_/_H5N1|2022-01-12
NP|A/goose/Czech_Republic/23458-1K/2021|EPI_ISL_9603780|A_/_H5N1|2021-11-26
NP|A/goose/Czech_Republic/23458-4T/2021|EPI_ISL_9603775|A_/_H5N1|2021-11-26
NP|A/goose/Czech_Republic/25322-229/2021|EPI_ISL_12325995|A_/_H5N1|2021-12-18
NP|A/goose/Czech_Republic/23458-2T/2021|EPI_ISL_9603767|A_/_H5N1|2021-11-26
NP|A/goose/Czech_Republic/25322-205/2021|EPI_ISL_12150666|A_/_H5N1|2021-12-18
NP|A/goose/Czech_Republic/23458-5T/2021|EPI_ISL_9603769|A_/_H5N1|2021-11-26
NP|A/goose/Czech_Republic/25322-179/2021|EPI_ISL_12150664|A_/_H5N1|2021-12-18
NP|A/goose/Czech_Republic/22750/2021|EPI_ISL_9603924|A_/_H5N1|2021-11-19
NP|A/goose/Czech_Republic/22608-1/2021|EPI_ISL_8515478|A_/_H5N1|2021-11-18
NP|A/goose/Czech_Republic/22608-2/2021|EPI_ISL_8515479|A_/_H5N1|2021-11-18
NP|A/goose/Czech_Republic/22608-3T/2021|EPI_ISL_9603901|A_/_H5N1|2021-11-18
NP|A/goose/Czech_Republic/22608-1T/2021|EPI_ISL_9603795|A_/_H5N1|2021-11-18
NP|A/goose/Czech_Republic/22608-2T/2021|EPI_ISL_9603819|A_/_H5N1|2021-11-18
NP|A/greylag_goose_/Sweden/SVA211118SZ0354/FB004497/I-2021|EPI_ISL_7055384|A_/_H5N1|2021-11-17
NP|A/swan/Slovenia/13_22VIR777-8/2022|EPI_ISL_11007535|A_/_H5N1|2022-01-03
NP|A/chicken/Netherlands/21038165-006010/2021|EPI_ISL_6101848|A_/_H5N1|2021-11-07
NP|A/Withe-tiled_eagle/Estonia/TA2124126-1_21VIR10433-11/2021|EPI_ISL_7778754|A_/_H5N1|2021-10-12
NP|A/chicken/Poland/H157_22VIR2515-3/2022|EPI_ISL_11922809|A_/_H5N1|2022-02-18
NP|A/domestic_duck/Poland/H1942-N/2021|EPI_ISL_6934175|A_/_H5N1|2021-11-07
NP|A/chicken/Czech_Republic/63/2022|EPI_ISL_11327090|A_/_H5N1|2022-01-03
NP|A/swan/Romania/10656_22VIR2749-6/2022|EPI_ISL_11922820|A_/_H5N1|2022-02-22
NP|A/greylag_goose/Netherlands/21037809-001/2021|EPI_ISL_6761000|A_/_H5N1|2021-10-31
NP|A/Anser_albifrons/Belgium/15465_0010/2021|EPI_ISL_9160206|A_/_H5N1|2021-11-21
NP|A/greylag_goose/Germany-SH/AI06144/2021|EPI_ISL_7753406|A_/_H5N1|2021-10-25
NP|A/mute_swan/Czech_Republic/21312/2021|EPI_ISL_6328409|A_/_H5N1|2021-11-01
NP|A/swan/France/21P012384/2021|EPI_ISL_6590766|A_/_H5N1|2021-11-08
NP|A/seagull/Italy/21VIR11259-10/2021|EPI_ISL_8882213|A_/_H5N1|2021-12-15
NP|A/mute_swan/Czech_Republic/25702-2/2021|EPI_ISL_9603927|A_/_H5N1|2021-12-20
NP|A/chicken/Czech_Republic/23404-2T/2021|EPI_ISL_7626512|A_/_H5N1|2021-11-25
NP|A/chicken/Czech_Republic/23404-4K/2021|EPI_ISL_7626506|A_/_H5N1|2021-11-25
NP|A/chicken/Czech_Republic/23404-4T/2021|EPI_ISL_7626513|A_/_H5N1|2021-11-25
NP|A/chicken/Czech_Republic/23404-2K/2021|EPI_ISL_7626484|A_/_H5N1|2021-11-25
NP|A/chicken/Czech_Republic/23404/2021|EPI_ISL_8515480|A_/_H5N1|2021-11-25
NP|A/swan/Romania/10324_22VIR2749-2/2022|EPI_ISL_11922816|A_/_H5N1|2022-02-01
NP|A/mute_swan/Czech_Republic/22477-1/2021|EPI_ISL_9603916|A_/_H5N1|2021-11-12
NP|A/mute_swan/Czech_Republic/22477-2/2021|EPI_ISL_9603918|A_/_H5N1|2021-11-12
NP|A/mute_swan/Czech_Republic/22684/2021|EPI_ISL_9603922|A_/_H5N1|2021-11-15
NP|A/mute_swan/Czech_Republic/2755/2022|EPI_ISL_12150661|A_/_H5N1|2022-01-29
NP|A/pheasant/Czech_Republic/25827-2/2021|EPI_ISL_9603937|A_/_H5N1|2021-12-25
NP|A/pheasant/Czech_Republic/25827-1/2021|EPI_ISL_9603931|A_/_H5N1|2021-12-25
NP|A/pheasant/Czech_Republic/25827-3/2021|EPI_ISL_9603939|A_/_H5N1|2021-12-25
NP|A/swan/Poland/MB083_22VIR2515-8/2022|EPI_ISL_11922814|A_/_H5N1|2022-02-17
0.02

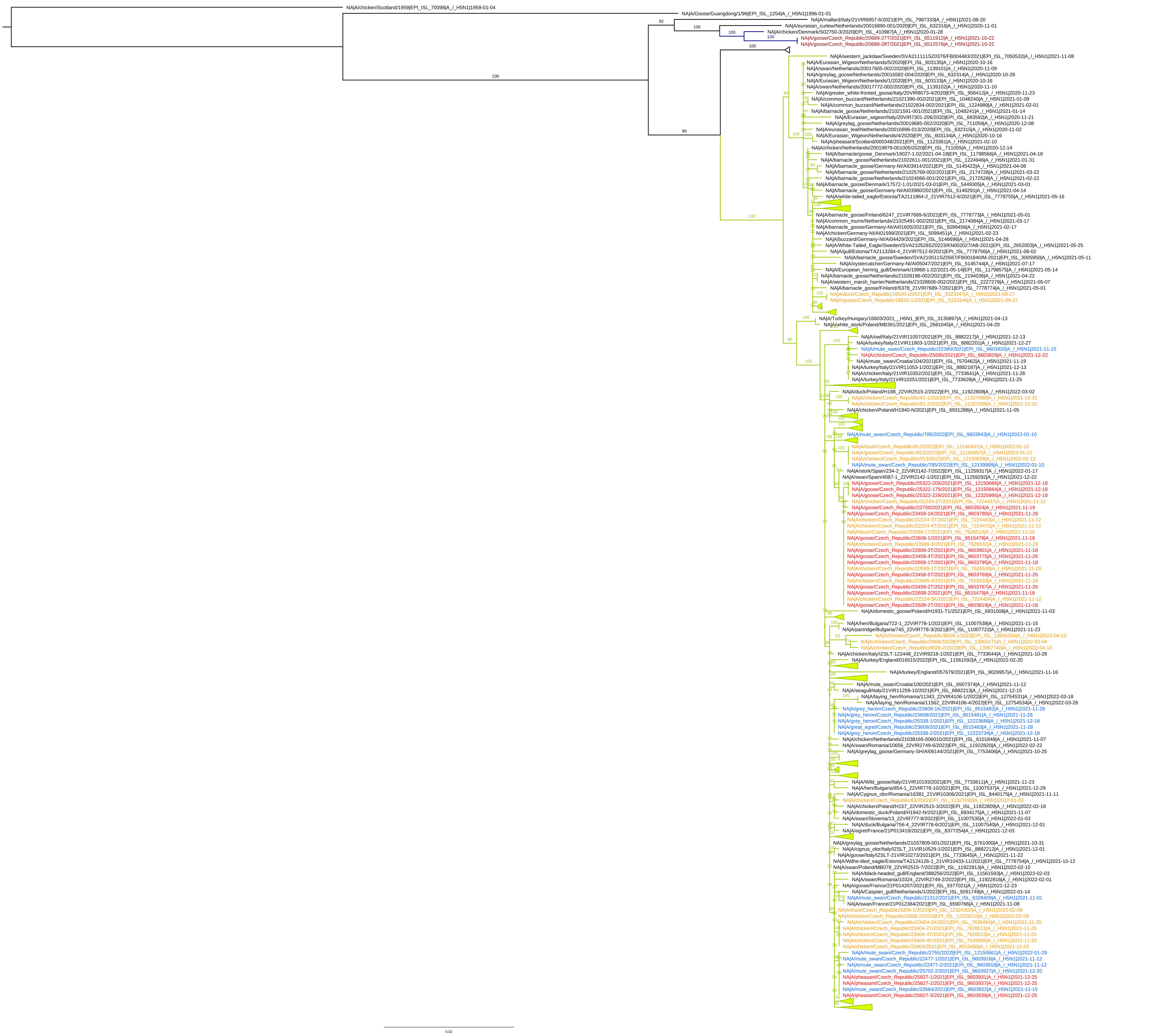

NA|A/chicken/Scotland/1959|EPI_ISL_70098|A_/_H5N1|1959-01-04
NA|A/Goose/Guangdong/1/96|EPI_ISL_1254|A_/_H5N1|1996-01-01
NA|A/mallard/Italy/21VIR6957-6/2021|EPI_ISL_7987333|A_/_H5N1|2021-08-20
NA|A/eurasian_curlew/Netherlands/20016890-001/2020|EPI_ISL_632316|A_/_H5N1|2020-11-01
NA|A/chicken/Denmark/S02750-3/2020|EPI_ISL_410987|A_/_H5N1|2020-01-28
NA|A/goose/Czech_Republic/20689-27T/2021|EPI_ISL_6511912|A_/_H5N1|2021-10-22
NA|A/goose/Czech_Republic/20689-28T/2021|EPI_ISL_6512576|A_/_H5N1|2021-10-22
NA|A/western_jackdaw/Sweden/SVA211111SZ0376/FB004483/2021|EPI_ISL_7050532|A_/_H5N1|2021-11-08
NA|A/Eurasian_Wigeon/Netherlands/5/2020|EPI_ISL_603135|A_/_H5N1|2020-10-16
NA|A/swan/Netherlands/20017605-002/2020|EPI_ISL_1139101|A_/_H5N1|2020-11-09
NA|A/greylag_goose/Netherlands/20016582-004/2020|EPI_ISL_632314|A_/_H5N1|2020-10-28
NA|A/Eurasian_Wigeon/Netherlands/1/2020|EPI_ISL_603133|A_/_H5N1|2020-10-16
NA|A/swan/Netherlands/20017772-002/2020|EPI_ISL_1139102|A_/_H5N1|2020-11-10
NA|A/greater_white-fronted_goose/Italy/20VIR8073-4/2020|EPI_ISL_956412|A_/_H5N1|2020-11-23
NA|A/common_buzzard/Netherlands/21021396-002/2021|EPI_ISL_1048240|A_/_H5N1|2021-01-09
NA|A/common_buzzard/Netherlands/21022834-002/2021|EPI_ISL_1224989|A_/_H5N1|2021-02-01
NA|A/barnacle_goose/Netherlands/21021591-001/2021|EPI_ISL_1048241|A_/_H5N1|2021-01-14
NA|A/Eurasian_wigeon/Italy/20VIR7301-206/2020|EPI_ISL_683592|A_/_H5N1|2020-11-21
NA|A/greylag_goose/Netherlands/20019685-002/2020|EPI_ISL_711059|A_/_H5N1|2020-12-08
NA|A/eurasian_teal/Netherlands/20016896-013/2020|EPI_ISL_632315|A_/_H5N1|2020-11-02
NA|A/Eurasian_Wigeon/Netherlands/4/2020|EPI_ISL_603134|A_/_H5N1|2020-10-16
NA|A/pheasant/Scotland/000348/2021|EPI_ISL_1123361|A_/_H5N1|2021-02-10
NA|A/chicken/Netherlands/20019879-001005/2020|EPI_ISL_711055|A_/_H5N1|2020-12-14
NA|A/barnacle/goose_Denmark/19027-1.02/2021-04-18|EPI_ISL_11798566|A_/_H5N1|2021-04-18
NA|A/barnacle_goose/Netherlands/21022611-001/2021|EPI_ISL_1224946|A_/_H5N1|2021-01-31
NA|A/barnacle_goose/Germany-NI/AI03914/2021|EPI_ISL_5145422|A_/_H5N1|2021-04-08
NA|A/barnacle_goose/Netherlands/21025769-002/2021|EPI_ISL_2174728|A_/_H5N1|2021-03-22
NA|A/barnacle_goose/Netherlands/21024066-001/2021|EPI_ISL_2172528|A_/_H5N1|2021-02-22
NA|A/barnacle_goose/Denmark/17572-1.01/2021-03-01|EPI_ISL_5449305|A_/_H5N1|2021-03-01
NA|A/barnacle_goose/Germany-NI/AI03980/2021|EPI_ISL_5146291|A_/_H5N1|2021-04-14
NA|A/white-tailed_eagle/Estonia/TA2111864-2_21VIR7512-6/2021|EPI_ISL_7778755|A_/_H5N1|2021-05-16
NA|A/barnacle_goose/Finland/6247_21VIR7689-6/2021|EPI_ISL_7778773|A_/_H5N1|2021-05-01
NA|A/common_murre/Netherlands/21025491-002/2021|EPI_ISL_2174084|A_/_H5N1|2021-03-17
NA|A/barnacle_goose/Germany-NI/AI01605/2021|EPI_ISL_5099456|A_/_H5N1|2021-02-17
NA|A/chicken/Germany-NI/AI01599/2021|EPI_ISL_5099451|A_/_H5N1|2021-02-23
NA|A/buzzard/Germany-NI/AI04429/2021|EPI_ISL_5146696|A_/_H5N1|2021-04-28
NA|A/White-Tailed_Eagle/Sweden/SVA210528SZ0223/KN002027/AB-2021|EPI_ISL_2652003|A_/_H5N1|2021-05-25
NA|A/gull/Estonia/TA2113284-4_21VIR7512-8/2021|EPI_ISL_7778756|A_/_H5N1|2021-06-02
NA|A/barnacle_goose/Sweden/SVA210511SZ0567/FB001840/M-2021|EPI_ISL_3005950|A_/_H5N1|2021-05-11
NA|A/oystercatcher/Germany-NI/AI05047/2021|EPI_ISL_5145744|A_/_H5N1|2021-07-17
NA|A/European_herring_gull/Denmark/19968-1.02/2021-05-14|EPI_ISL_11798575|A_/_H5N1|2021-05-14
NA|A/barnacle_goose/Netherlands/21028196-002/2021|EPI_ISL_2194036|A_/_H5N1|2021-04-22
NA|A/western_marsh_harrier/Netherlands/21028606-002/2021|EPI_ISL_2227279|A_/_H5N1|2021-05-07
NA|A/barnacle_goose/Finland//6378_21VIR7689-7/2021|EPI_ISL_7778774|A_/_H5N1|2021-05-01
NA|A/duck/Czech_Republic/18520-2/2021|EPI_ISL_5323347|A_/_H5N1|2021-09-27
NA|A/goose/Czech_Republic/18520-1/2021|EPI_ISL_5323346|A_/_H5N1|2021-09-27
NA|A/Turkey/Hungary/16603/2021__H5N1_|EPI_ISL_3135897|A_/_H5N1|2021-04-13
NA|A/white_stork/Poland/MB391/2021|EPI_ISL_2681045|A_/_H5N1|2021-04-20
NA|A/owl/Italy/21VIR11057/2021|EPI_ISL_8882217|A_/_H5N1|2021-12-13
NA|A/turkey/Italy/21VIR11803-1/2021|EPI_ISL_8882201|A_/_H5N1|2021-12-27
NA|A/mute_swan/Czech_Republic/22380/2021|EPI_ISL_9603920|A_/_H5N1|2021-11-15
NA|A/chicken/Czech_Republic/25690/2021|EPI_ISL_9603929|A_/_H5N1|2021-12-22
NA|A/mute_swan/Croatia/104/2021|EPI_ISL_7570462|A_/_H5N1|2021-11-19
NA|A/turkey/Italy/21VIR11053-1/2021|EPI_ISL_8882187|A_/_H5N1|2021-12-13
NA|A/chicken/Italy/21VIR10352/2021|EPI_ISL_7733641|A_/_H5N1|2021-11-26
NA|A/turkey/Italy/21VIR10251/2021|EPI_ISL_7733628|A_/_H5N1|2021-11-25
NA|A/duck/Poland/H188_22VIR2515-2/2022|EPI_ISL_11922808|A_/_H5N1|2022-03-02
NA|A/chicken/Czech_Republic/61-1/2022|EPI_ISL_11327088|A_/_H5N1|2021-12-31
NA|A/chicken/Czech_Republic/61-2/2022|EPI_ISL_11327089|A_/_H5N1|2021-12-31
NA|A/chicken/Poland/H1940-N/2021|EPI_ISL_6931288|A_/_H5N1|2021-11-05
NA|A/mute_swan/Czech_Republic/785/2022|EPI_ISL_9603943|A_/_H5N1|2022-01-10
NA|A/duck/Czech_Republic/913/2022|EPI_ISL_12140437|A_/_H5N1|2022-01-12
NA|A/goose/Czech_Republic/913/2022|EPI_ISL_12150657|A_/_H5N1|2022-01-12
NA|A/chicken/Czech_Republic/913/2022|EPI_ISL_12150658|A_/_H5N1|2022-01-12
NA|A/mute_swan/Czech_Republic/785/2022|EPI_ISL_12139999|A_/_H5N1|2022-01-10
NA|A/stork/Spain/234-2_22VIR2142-7/2022|EPI_ISL_11259317|A_/_H5N1|2022-01-17
NA|A/swan/Spain/4087-1_22VIR2142-1/2021|EPI_ISL_11259292|A_/_H5N1|2021-12-22
NA|A/goose/Czech_Republic/25322-205/2021|EPI_ISL_12150666|A_/_H5N1|2021-12-18
NA|A/goose/Czech_Republic/25322-179/2021|EPI_ISL_12150664|A_/_H5N1|2021-12-18
NA|A/goose/Czech_Republic/25322-229/2021|EPI_ISL_12325995|A_/_H5N1|2021-12-18
NA|A/chicken/Czech_Republic/22224-2T/2021|EPI_ISL_7224437|A_/_H5N1|2021-11-12
NA|A/goose/Czech_Republic/22750/2021|EPI_ISL_9603924|A_/_H5N1|2021-11-19
NA|A/goose/Czech_Republic/23458-1K/2021|EPI_ISL_9603780|A_/_H5N1|2021-11-26
NA|A/chicken/Czech_Republic/22224-3T/2021|EPI_ISL_7224463|A_/_H5N1|2021-11-12
NA|A/chicken/Czech_Republic/22224-4T/2021|EPI_ISL_7224472|A_/_H5N1|2021-11-12
NA|A/duck/Czech_Republic/23589-1T/2021|EPI_ISL_7626514|A_/_H5N1|2021-11-29
NA|A/goose/Czech_Republic/22608-1/2021|EPI_ISL_8515478|A_/_H5N1|2021-11-18
NA|A/chicken/Czech_Republic/23589-3/2021|EPI_ISL_7626532|A_/_H5N1|2021-11-29
NA|A/goose/Czech_Republic/22608-3T/2021|EPI_ISL_9603901|A_/_H5N1|2021-11-18
NA|A/goose/Czech_Republic/23458-4T/2021|EPI_ISL_9603775|A_/_H5N1|2021-11-26
NA|A/goose/Czech_Republic/22608-1T/2021|EPI_ISL_9603795|A_/_H5N1|2021-11-18
NA|A/chicken/Czech_Republic/23589-1T/2021|EPI_ISL_7626526|A_/_H5N1|2021-11-29
NA|A/goose/Czech_Republic/23458-5T/2021|EPI_ISL_9603769|A_/_H5N1|2021-11-26
NA|A/chicken/Czech_Republic/23589-4/2021|EPI_ISL_7626533|A_/_H5N1|2021-11-29
NA|A/goose/Czech_Republic/23458-2T/2021|EPI_ISL_9603767|A_/_H5N1|2021-11-26
NA|A/goose/Czech_Republic/22608-2/2021|EPI_ISL_8515479|A_/_H5N1|2021-11-18
NA|A/chicken/Czech_Republic/22224-3K/2021|EPI_ISL_7224454|A_/_H5N1|2021-11-12
NA|A/goose/Czech_Republic/22608-2T/2021|EPI_ISL_9603819|A_/_H5N1|2021-11-18
NA|A/domestic_goose/Poland/H1931-T1/2021|EPI_ISL_6931008|A_/_H5N1|2021-11-03
NA|A/hen/Bulgaria/722-1_22VIR778-1/2021|EPI_ISL_11007538|A_/_H5N1|2021-11-15
NA|A/partridge/Bulgaria/745_22VIR778-3/2021|EPI_ISL_11007722|A_/_H5N1|2021-11-23
NA|A/chicken/Czech_Republic/8028-1/2022|EPI_ISL_13955204|A_/_H5N1|2022-04-13
NA|A/chicken/Czech_Republic/2968/2022|EPI_ISL_13955171|A_/_H5N1|2022-02-04
NA|A/chicken/Czech_Republic/8028-2/2022|EPI_ISL_13967740|A_/_H5N1|2022-04-13
NA|A/chicken/Italy/IZSLT-122448_21VIR9218-1/2021|EPI_ISL_7733644|A_/_H5N1|2021-10-28
NA|A/turkey/England/016515/2022|EPI_ISL_11561592|A_/_H5N1|2022-02-20
NA|A/turkey/England/057679/2021|EPI_ISL_9029957|A_/_H5N1|2021-11-16
NA|A/mute_swan/Croatia/100/2021|EPI_ISL_6507374|A_/_H5N1|2021-11-12
NA|A/seagull/Italy/21VIR11259-10/2021|EPI_ISL_8882213|A_/_H5N1|2021-12-15
NA|A/laying_hen/Romania/11343_22VIR4106-1/2022|EPI_ISL_12754531|A_/_H5N1|2022-03-18
NA|A/laying_hen/Romania/11562_22VIR4106-4/2022|EPI_ISL_12754534|A_/_H5N1|2022-03-26
NA|A/grey_heron/Czech_Republic/23608-1K/2021|EPI_ISL_8515482|A_/_H5N1|2021-11-28
NA|A/grey_heron/Czech_Republic/23608/2021|EPI_ISL_8515481|A_/_H5N1|2021-11-28
NA|A/grey_heron/Czech_Republic/25338-1/2021|EPI_ISL_12223688|A_/_H5N1|2021-12-18
NA|A/great_egret/Czech_Republic/23609/2021|EPI_ISL_8515483|A_/_H5N1|2021-11-28
NA|A/grey_heron/Czech_Republic/25338-2/2021|EPI_ISL_12223734|A_/_H5N1|2021-12-18
NA|A/chicken/Netherlands/21038165-006010/2021|EPI_ISL_6101848|A_/_H5N1|2021-11-07
NA|A/swan/Romania/10656_22VIR2749-6/2022|EPI_ISL_11922820|A_/_H5N1|2022-02-22
NA|A/greylag_goose/Germany-SH/AI06144/2021|EPI_ISL_7753406|A_/_H5N1|2021-10-25
NA|A/Wild_goose/Italy/21VIR10193/2021|EPI_ISL_7733611|A_/_H5N1|2021-11-23
NA|A/hen/Bulgaria/854-1_22VIR778-10/2021|EPI_ISL_11007537|A_/_H5N1|2021-12-29
NA|A/Cygnus_olor/Romania/16381_21VIR10306/2021|EPI_ISL_8440175|A_/_H5N1|2021-11-11
NA|A/chicken/Czech_Republic/63/2022|EPI_ISL_11327090|A_/_H5N1|2022-01-03
NA|A/chicken/Poland/H157_22VIR2515-3/2022|EPI_ISL_11922809|A_/_H5N1|2022-02-18
NA|A/domestic_duck/Poland/H1942-N/2021|EPI_ISL_6934175|A_/_H5N1|2021-11-07
NA|A/swan/Slovenia/13_22VIR777-8/2022|EPI_ISL_11007535|A_/_H5N1|2022-01-03
NA|A/duck/Bulgaria/756-4_22VIR778-6/2021|EPI_ISL_11007540|A_/_H5N1|2021-12-01
NA|A/egret/France/21P013418/2021|EPI_ISL_8377254|A_/_H5N1|2021-12-03
NA|A/greylag_goose/Netherlands/21037809-001/2021|EPI_ISL_6761000|A_/_H5N1|2021-10-31
NA|A/cignus_olor/Italy/IZSLT_21VIR10529-1/2021|EPI_ISL_8882212|A_/_H5N1|2021-12-01
NA|A/goose/Italy/IZSLT-21VIR10273/2021|EPI_ISL_7733645|A_/_H5N1|2021-11-22
NA|A/Withe-tiled_eagle/Estonia/TA2124126-1_21VIR10433-11/2021|EPI_ISL_7778754|A_/_H5N1|2021-10-12
NA|A/swan/Poland/MB078_22VIR2515-7/2022|EPI_ISL_11922813|A_/_H5N1|2022-02-10
NA|A/black-headed_gull/England/388256/2022|EPI_ISL_11561593|A_/_H5N1|2022-02-03
NA|A/swan/Romania/10324_22VIR2749-2/2022|EPI_ISL_11922816|A_/_H5N1|2022-02-01
NA|A/goose/France/21P014207/2021|EPI_ISL_9377021|A_/_H5N1|2021-12-23
NA|A/Caspian_gull/Netherlands/1/2022|EPI_ISL_9261749|A_/_H5N1|2022-01-14
NA|A/mute_swan/Czech_Republic/21312/2021|EPI_ISL_6328409|A_/_H5N1|2021-11-01
NA|A/swan/France/21P012384/2021|EPI_ISL_6590766|A_/_H5N1|2021-11-08
NA|A/duck/Czech_Republic/3306-1/2022|EPI_ISL_12324302|A_/_H5N1|2022-02-09
NA|A/chicken/Czech_Republic/3306-2/2022|EPI_ISL_12325210|A_/_H5N1|2022-02-09
NA|A/chicken/Czech_Republic/23404-2K/2021|EPI_ISL_7626484|A_/_H5N1|2021-11-25
NA|A/chicken/Czech_Republic/23404-2T/2021|EPI_ISL_7626512|A_/_H5N1|2021-11-25
NA|A/chicken/Czech_Republic/23404-4T/2021|EPI_ISL_7626513|A_/_H5N1|2021-11-25
NA|A/chicken/Czech_Republic/23404-4K/2021|EPI_ISL_7626506|A_/_H5N1|2021-11-25
NA|A/chicken/Czech_Republic/23404/2021|EPI_ISL_8515480|A_/_H5N1|2021-11-25
NA|A/mute_swan/Czech_Republic/2755/2022|EPI_ISL_12150661|A_/_H5N1|2022-01-29
NA|A/mute_swan/Czech_Republic/22477-1/2021|EPI_ISL_9603916|A_/_H5N1|2021-11-12
NA|A/mute_swan/Czech_Republic/22477-2/2021|EPI_ISL_9603918|A_/_H5N1|2021-11-12
NA|A/mute_swan/Czech_Republic/25702-2/2021|EPI_ISL_9603927|A_/_H5N1|2021-12-20
NA|A/pheasant/Czech_Republic/25827-1/2021|EPI_ISL_9603931|A_/_H5N1|2021-12-25
NA|A/pheasant/Czech_Republic/25827-2/2021|EPI_ISL_9603937|A_/_H5N1|2021-12-25
NA|A/mute_swan/Czech_Republic/22684/2021|EPI_ISL_9603922|A_/_H5N1|2021-11-15
NA|A/pheasant/Czech_Republic/25827-3/2021|EPI_ISL_9603939|A_/_H5N1|2021-12-25
0.02

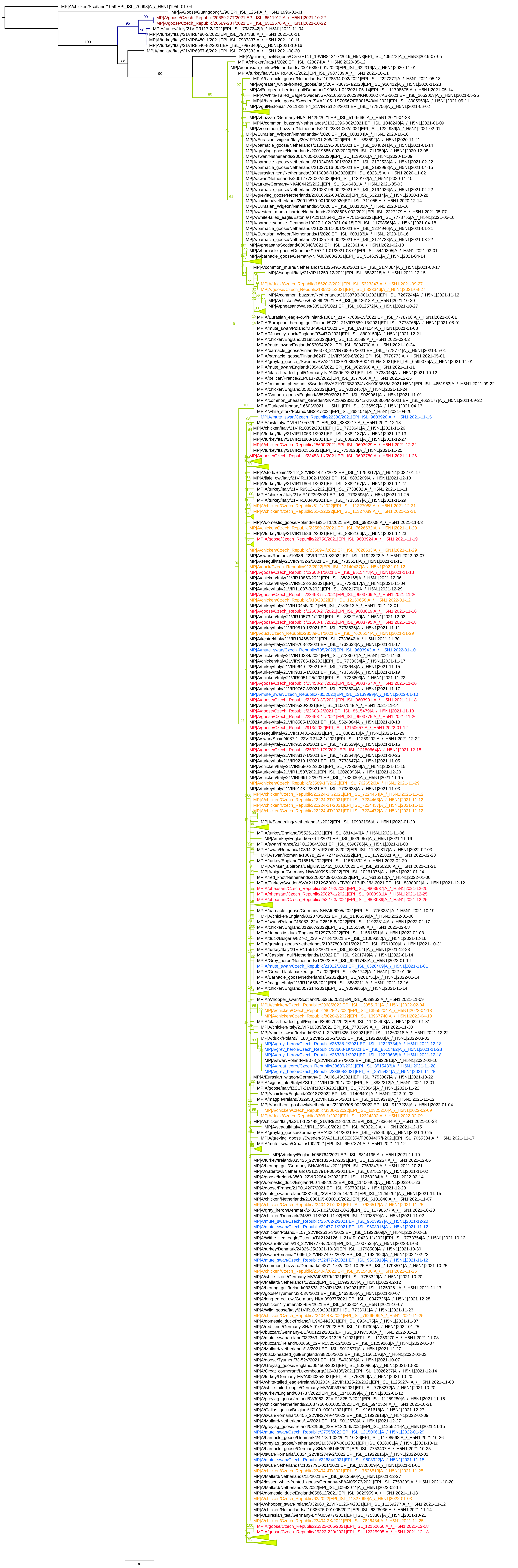

0.008

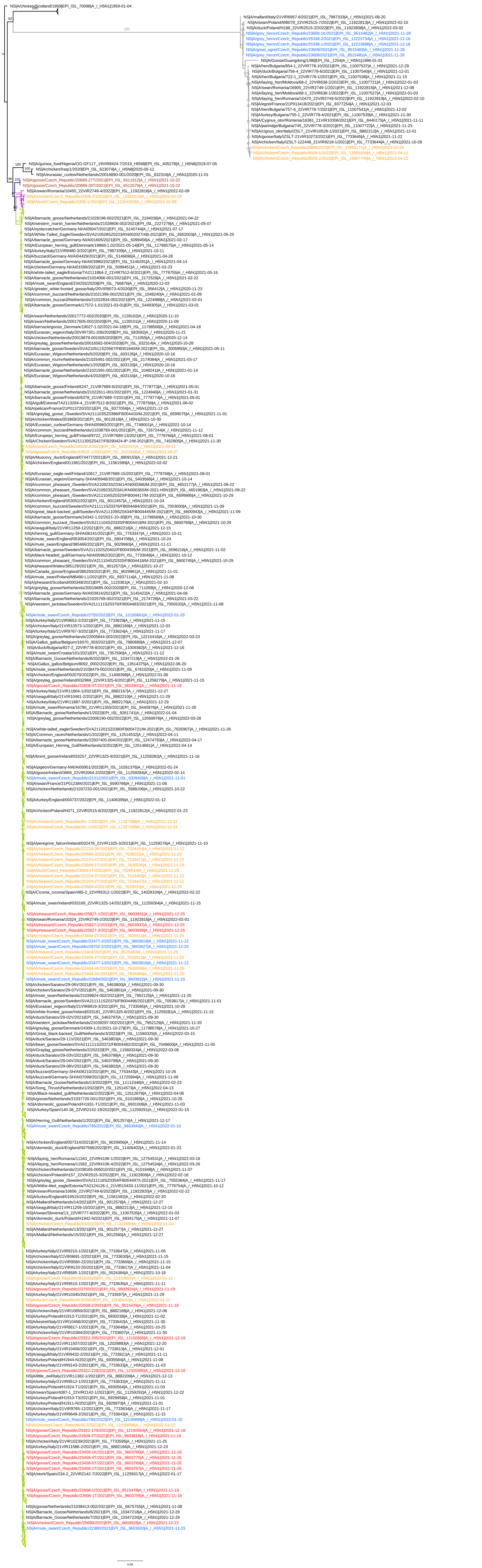

Supplementary Material 1, Figure S1. Phylogenetic analysis of Czech H5N1 HP/LPAI strains detected during the 2021/2022 influenza season.

The ML tree (Best-fit substitution models according to Bayesian information criterion: PB2: GTR+F+G4; PB1: UNREST+FO+I+G4; PA: UNREST+FO+G4; H5: GTR+F+G4; NP: GTR+F+I+G4; N1: TVM+F+G4; MP: K3P+G4; NS: TVM+F+I+G4) was calculated separately for each genomic segment, based on Eurasian H5N1 sequences collected between September 2021 and Jun 2022 and stored in the GISAID EpiFlu database PB2 n=602, PB1 n=591, PA=593, H5 n=661, NP=590, N1 n=594, MP n=599 and NS n=597. Colouring: red-commercial, orange-backyard, and blue-wild. For each branch, the bootstrap values (1000 replicates) in percentages are indicated. All trees except N1 were rooted to A/chicken/Scotland/1959 H5N1. The N1 tree was rooted to A/goose/Guangdong/1/1996. The branches of the tree corresponding with discrete genotypes were highlighted with a genotype specific colour (Figure 3).

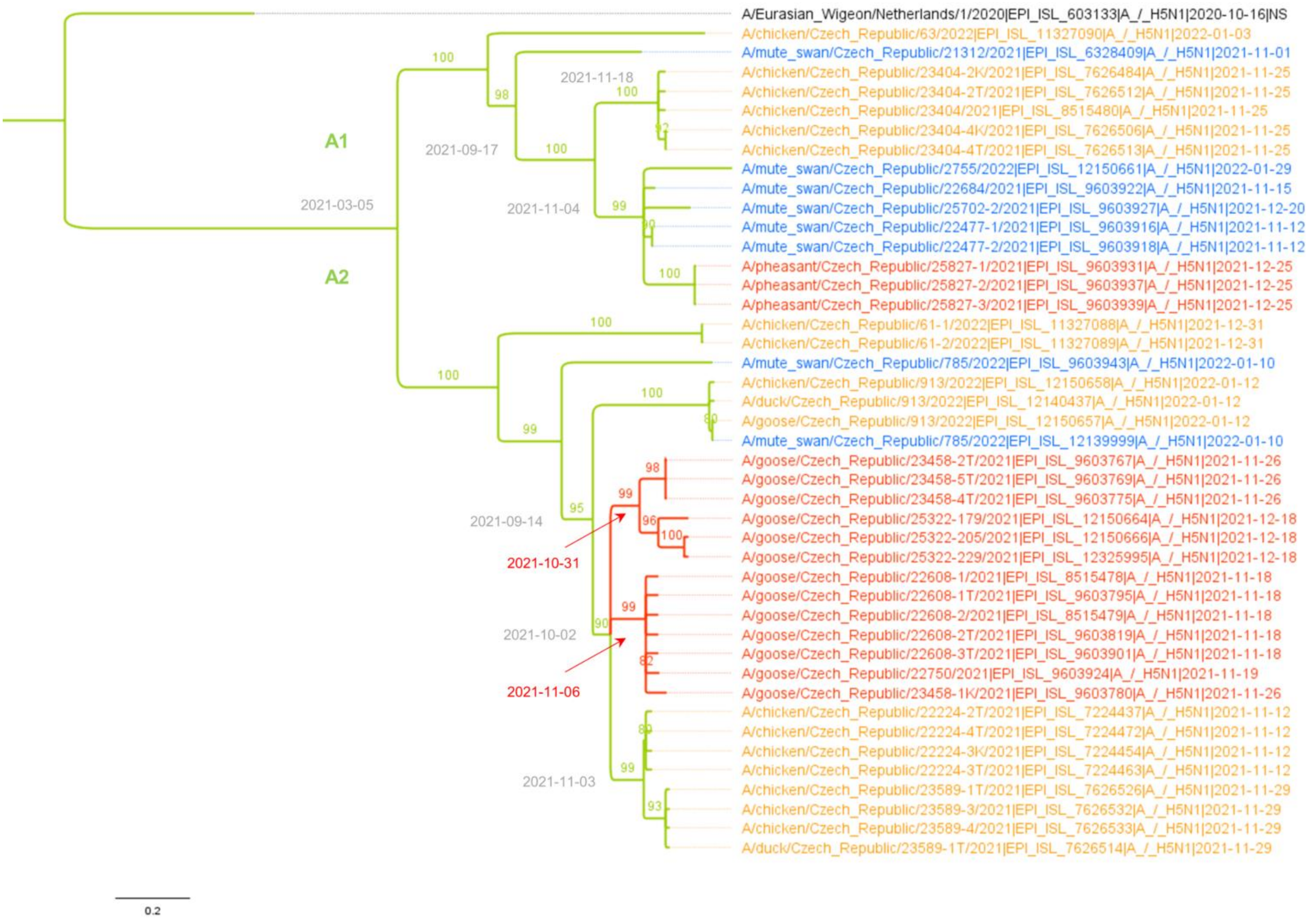

Supplementary Material 1, Figure S2. Phylogenetic dating.

Concatenated ML tree with the most probable divergence times estimated using the LSD approach. The tree was rooted to the A/ew/NL/1/2020 H5N1 strain. For clarity, only the subtree encompassing genotypes A1 and A2 (Figure 3) was provided. The order of segments in the concatenated genomes is PB2, PB1, PA, H5, NP, N1, MP and NS. The tree was computed using IQ-TREE (GTR+F+R2 as the best fitting model selected according to the Bayesian information criterion) and scaled with branch lengths measured in number of substitutions per site. For each branch, bootstrap values (1000 replicates) were given in percentages. The branches consisted of H5N1 HPAI strains from geese are highlighted red. Virus taxa were highlighted according to origin: red-commercial; orange-backyard; and blue-wild.
